# High LRRK2 Levels Fail to Induce or Exacerbate Neuronal Alpha-Synucleinopathy in Mouse Brain

**DOI:** 10.1371/journal.pone.0036581

**Published:** 2012-05-15

**Authors:** Martin C. Herzig, Michael Bidinosti, Tatjana Schweizer, Thomas Hafner, Christine Stemmelen, Andreas Weiss, Simone Danner, Nella Vidotto, Daniela Stauffer, Carmen Barske, Franziska Mayer, Peter Schmid, Giorgio Rovelli, P. Herman van der Putten, Derya R. Shimshek

**Affiliations:** Department of Neuroscience, Novartis Institutes for BioMedical Research, Novartis Pharma AG, Basel, Switzerland; UCL Institute of Neurology, United Kingdom

## Abstract

The G2019S mutation in the multidomain protein leucine-rich repeat kinase 2 (LRRK2) is one of the most frequently identified genetic causes of Parkinson’s disease (PD). Clinically, LRRK2(G2019S) carriers with PD and idiopathic PD patients have a very similar disease with brainstem and cortical Lewy pathology (α-synucleinopathy) as histopathological hallmarks. Some patients have Tau pathology. Enhanced kinase function of the LRRK2(G2019S) mutant protein is a prime suspect mechanism for carriers to develop PD but observations in LRRK2 knock-out, G2019S knock-in and kinase-dead mutant mice suggest that LRRK2 steady-state abundance of the protein also plays a determining role. One critical question concerning the molecular pathogenesis in LRRK2(G2019S) PD patients is whether α-synuclein (aSN) has a contributory role. To this end we generated mice with high expression of either wildtype or G2019S mutant LRRK2 in brainstem and cortical neurons. High levels of these LRRK2 variants left endogenous aSN and Tau levels unaltered and did not exacerbate or otherwise modify α-synucleinopathy in mice that co-expressed high levels of LRRK2 and aSN in brain neurons. On the contrary, in some lines high LRRK2 levels improved motor skills in the presence and absence of aSN-transgene-induced disease. Therefore, in many neurons high LRRK2 levels are well tolerated and not sufficient to drive or exacerbate neuronal α-synucleinopathy.

## Introduction

Parkinson’s disease (PD) is a common neurodegenerative movement disorder with clinical features including bradykinesia, rigidity and resting tremor. PD histopathological hallmarks are the loss of dopaminergic neurons in the substantia nigra and Lewy pathology. The latter is characterized by fibrillar α-synuclein (aSN) aggregates that are microscopically visible and referred to as Lewy bodies (LB) and Lewy neurites. This α-synuclein proteinopathy is often widespread and affects not only dopaminergic neurons in the substantia nigra but also neurons in other brainstem nuclei, the cortex, the spinal cord and the gastrointestinal nervous system [1], [2], [3].

The first mutation causing PD was discovered in the aSN-encoding gene (SNCA) [4]. Since then, many more PD loci including leucine-rich repeat kinase-2 (LRRK2) have been discovered through linkage analysis or genome-wide association studies (GWAS) [5], [6], [7], [8], [9], [10], [11], [12], [13]. Furthermore, polymorphic variants of genes including SNCA, LRRK2 and microtubule-associated protein Tau (MAPT) have emerged as susceptibility factors associated with an increased risk to develop PD [14], [15], [16], [17], [18].

LRRK2 mutations cause late-onset autosomal dominant PD that is clinically indistinguishable from idiopathic PD. They account for approx. 4–5% of familiar and 1–2% of sporadic PD [5], [6], [7], [8], [9], [10], [19], [20]. In addition, LRRK2 has been implicated as a susceptibility factor in other diseases like Crohn’s disease [21], [22], [23], cancer [24], [25] and leprosy [26] which could suggest unrecognized links between these disease pathophysiologies [27]. The most prominent PD-associated mutation G2019S was shown to result in increased kinase activity [28], [29], [30] and induce neuronal toxicity [31], [32], [33]. Such findings support the hypothesis that enhanced LRRK2 kinase function might suffice to evoke neuropathophysiological changes. They also raised hope that LRRK2 kinase inhibitors might be capable of halting disease progression in LRRK2(G2019S) and perhaps other LRRK2 mutation carriers. Although the enhanced kinase function of the LRRK2(G2019S) mutant is the prime suspect mechanism for carriers with this mutation to develop PD, the discovery by us and others of LRRK2-dependent phenotypes in kidney suggest that also steady-state abundance of the LRRK2 protein might play a determining role [30], [34].

In patients with LRRK2 mutations and clinically manifest PD, the associated neuropathology is heterogeneous ranging from LB pathology with a variable burden of Tau neurofibrillary tangles (NFTs) to Tau-only pathology and no inclusions [10], [35], [36], [37], [38]. R1441C carriers seem to lack LB pathology and an initial report described pathological variability even within the same family [10]. In contrast, most autopsies of LRRK2(G2019S) mutation carriers with PD show LB pathology (e.g. 19/22, [39]; 3/3, [40]) although the brain regions displaying Lewy pathology are variable. For example, extensive cortical involvement was reported in 7/19 cases whereas 12 out of the same 19 cases had extensive brainstem pathology ([40] and discussion therein). So genetic factors other than LRRK2 itself and environmental risk factors might exacerbate PD related changes in LRRK2(G2019S) patients and determine also the extent of cortical involvement.

One critical question concerning the molecular pathogenesis in LRRK2(G2019S) PD patients is whether the SNCA gene and aSN protein levels have a contributory role. It is known, for example, that expansion of SNCA Rep1, an upstream polymorphic microsatellite of the SNCA gene, is associated with elevated risk for sporadic PD [41], [42], [43]. Also, Rep1 regulates SNCA expression by enhancing its transcription in the nervous system [44], [45]. So far, human genetic data have not been disclosed to suggest synergistic effects in PD pathogenesis of the LRRK2(G2019S) allele and aSN levels as dictated by SNCA gene polymorphisms. In mice, two groups have reported co- and over-expression of LRRK2 and aSN [46], [47]. Lin et al. [47] showed that under control of a strongly forebrain-selective CaMKII-tTA promoter, over-expression of a tetO-LRRK2(WT) or tetO-LRRK2(G2019S) transgene failed to cause neurodegeneration. However, both LRRK2 variants accelerated the progression of a tetO-aSN transgene-mediated neuronal loss and α-synucleinopathy in CaMKII-tTA/tetO-LRRK2/tetO-aSN transgenic mice and exacerbated the accompanying astrocytosis and microgliosis. The most prominent effects were reported in striatum but cortex was also affected. These findings contrast with recent results reported by Daher et al. [46]. Co-expression of aSN(A53T) and LRRK2(G2019S) under control of a hindbrain selective prion promoter had minimal impact on the lethal neurodegenerative phenotype and predominantly hindbrain-selective α-synuclein-related pathology that developed in the aSN(A53T) mice. The latter study therefore failed to provide support for a pathophysiological interaction of LRRK2 and aSN in mouse hindbrain neurons. Here, we experimentally approached this question differently by generating double transgenic mice co-expressing under the control of mouse Thy1-regulatory sequences high levels of aSN and LRRK2 in a large population of both forebrain and brainstem neurons.

## Results and Discussion

**Figure 1:**
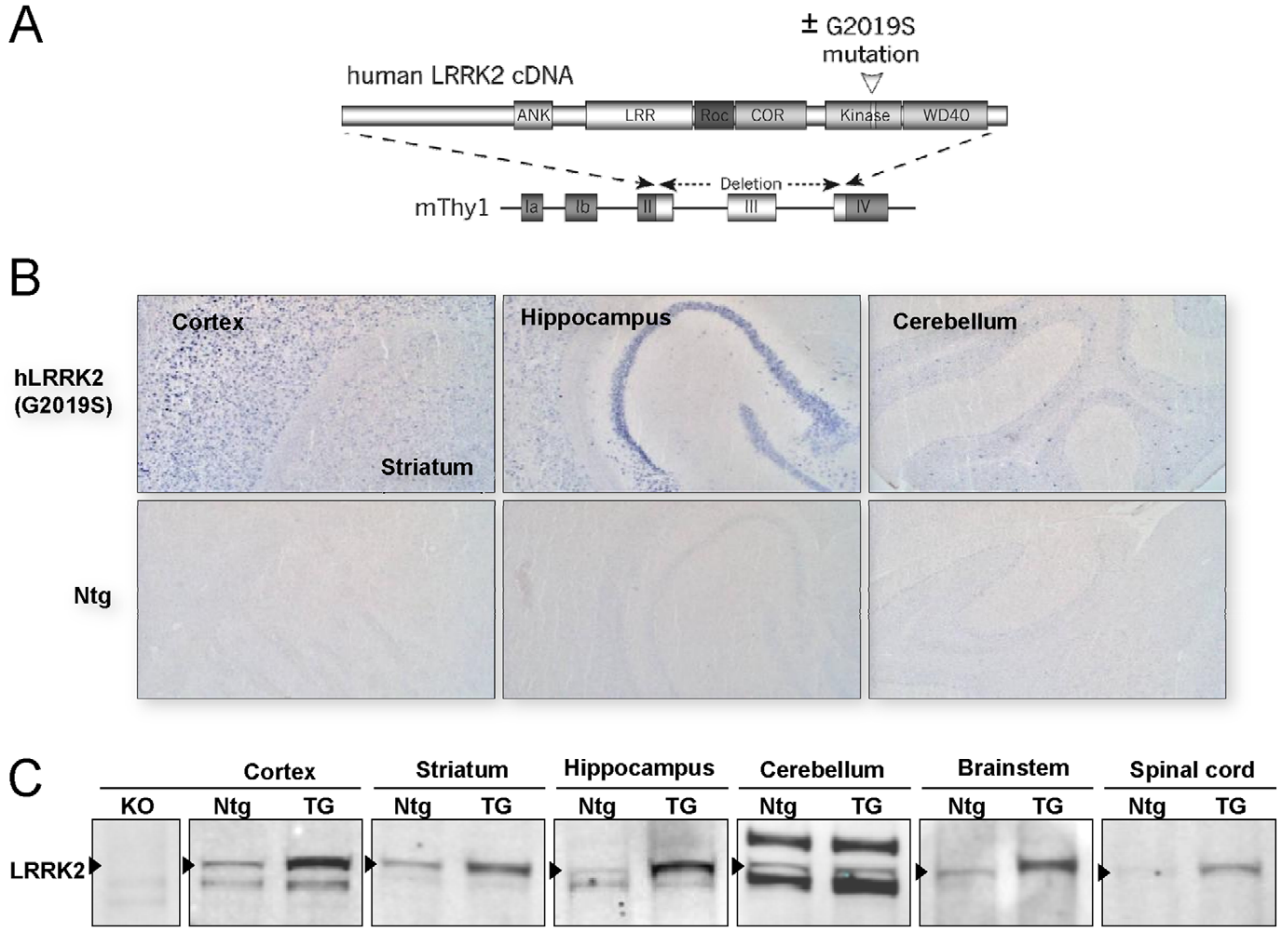
hLRRK2(G2019S) transgene mRNA and protein expression in the mouse brain. (A) Schematic representation of wildtype and G2019S-mutant human LRRK2-encoding cDNA inserted into the murine Thy1 expression cassette (mThy1). (B) Transgene hLRRK2(G2019S) mRNA expression pattern comparing transgenic and Ntg mouse brain regions and visualized using a DIG-labeled cDNA probe. (C) Immunoblots showing expression of endogenous and transgene LRRK2 protein in different brain regions of Ntg and TG [hLRRK2(G2019S)] mice. Note, LRRK2 is indicated by arrowheads and dependent on the brain region, different unspecific cross-reacting proteins are detected as well. LRRK2 knock-out (KO) cortex is included as a negative control. Ntg: non-transgenic wildtype littermate control.

To analyze whether increased levels of LRRK2 compromise neuronal integrity *in vivo* and trigger endogenous aSN-gene-driven or exacerbate transgene-driven α-synucleinopathy, we generated mouse lines that over-express human wildtype LRRK2 (hLRRK2(WT)) or the PD-associated G2019S mutant (hLRRK2(G2019S)). Both LRRK2 cDNAs were expressed under the control of Thy1 regulatory sequences which direct widespread expression in neurons in cortex, brainstem and spinal cord (**Figure 1A** and [48], [49], [50]). Out of five hLRRK2(WT) lines, four lines showed either no or variable transgene expression. One line selected for further studies showed strong and stable LRRK2 over-expression (**Figure S1** and data not shown). From seven hLRRK2(G2019S) founders, we obtained two lines with high and stable LRRK2 expression (**Figure 1C**,**S1** and data not shown). One of the latter lines and the one hLRRK2(WT) line were used for further studies.

*In situ* hybridizations using an anti-sense riboprobe corresponding to nucleotides 3537–4024 of the human LRRK2 transcript (**Figure 1B**,[Supplementary-material pone.0036581.s002] **S2**) and Western blot analysis (**Figure 1C**,**S1**) confirmed high and widespread expression of the LRRK2 transgene in all brain areas, except for the cerebellum (**Figure 1B,1C,S** **1,S2**). Protein and transgene expression levels of the hLRRK2(WT) and hLRRK2(G2019S) transgenes were very similar in cortex, hippocampus and brainstem (**Figure S1,S2**) but slightly lower for the former in spinal cord (**Figure S1**) and striatum (**Figure S1,S2**).

No obvious brain pathology developed in either line up to the age of 19 months (oldest age analyzed; data not shown). Others have reported similar negative findings in αCamKII-LRRK2 transgenics [47] whereas high LRRK2 levels targeted to the dopaminergic neurons seem to affect their integrity and viability [33], [51], [52]. We could not assess this in our lines because like most Thy1 based lines, also our lines lack expression in substantia nigra dopaminergic neurons. An attempt to generate lines expressing LRRK2 under control of the tyrosine hydroxylase promoter failed (data not shown).

**Figure 2:**
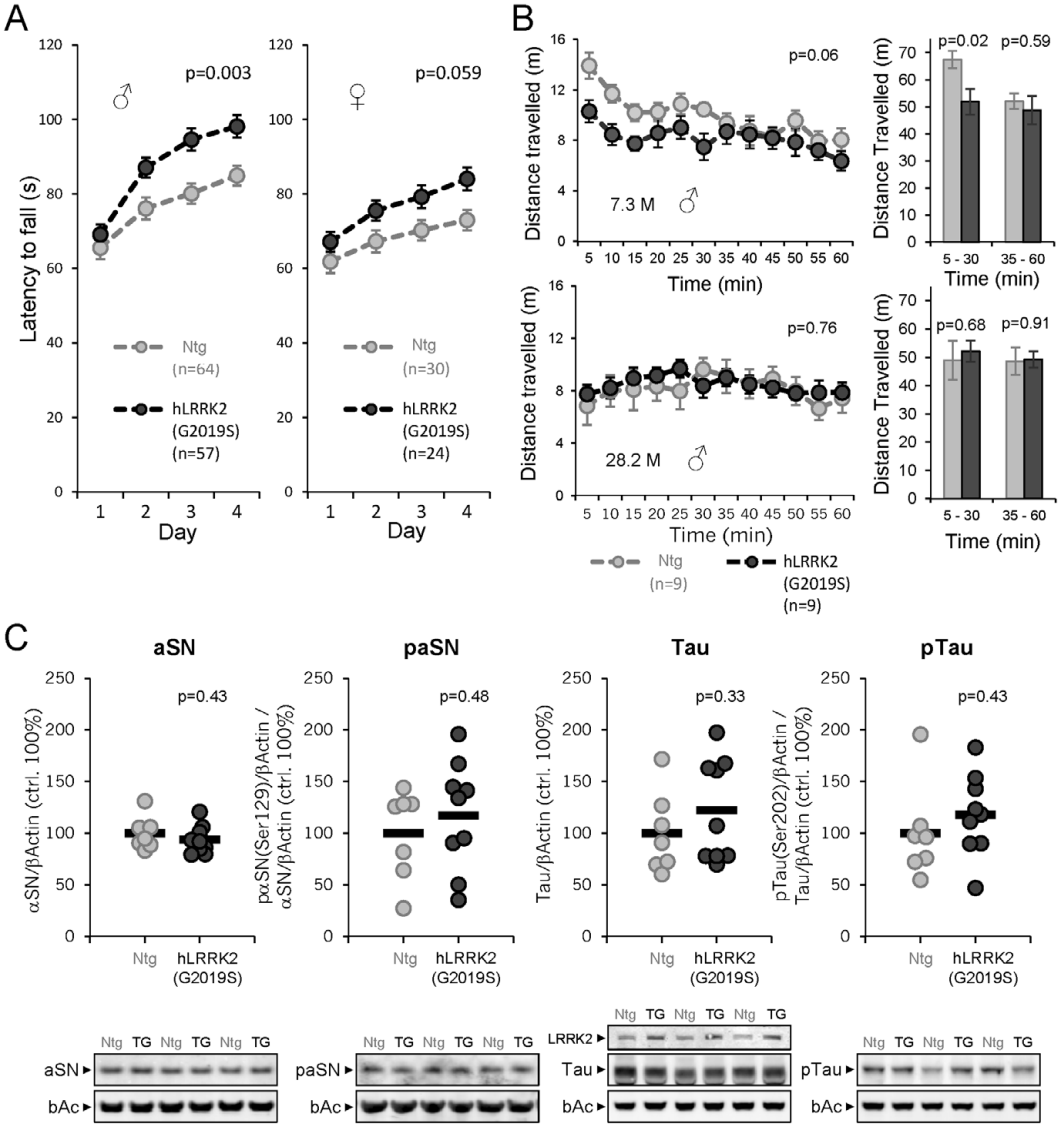
Motor assessment and aSN/Tau protein characterization in hLRRK2(G2019S) mice. (A) Motor skill learning of 4-month-old male and 6-month-old female hLRRK2(G2019S) and Ntg controls in the 3-step accelerated rotarod task over four consecutive days. The number of mice per genotype is indicated. Three batches of animals were included in this graph (single transgenic and Ntg animals from experiments shown in Figure 3B as well as a separate batch). p-values were determined by repeated measure ANOVA (group effect males: F(1,119) = 9.42, p = 0.003, group effect females: F(1,52) = 3.74, p = 0.059). (B) Novelty-induced horizontal locomotor activity of 7.3- and 28.2-month-old hLRRK2(G2019S) and Ntg mice. Bar graphs show the sum of the distance travelled from 5–30 min and from 35–60 min. The number of mice per genotype is indicated. p-values were determined either by repeated measure ANOVA (group effect males 7.3 M: F(1,16) = 4.044, p = 0.061; group effect males 28.2 M: F(1,16) = 0.093, p = 0.764) or by two-tailed, unequal variances Student’s t-test. (C) Western blotting of forebrain homogenates from 15-month-old hLRRK2(G2019S) (TG) and Ntg male mice. Lower panel: Shown are levels of mouse α-synuclein (aSN) and phospho-α-synuclein Ser129 (paSN) as well as mouse microtubule-associated protein Tau and phospho-Tau Ser202/Thr205 (pTau). β-actin (βAc) was used as loading control and for normalization. Upper panel shows the results of the immunoblot quantifications. Circles represent individual mice, the means (% normalized to Ntg) are indicated as horizontal bars. p-values were determined by two-tailed, unequal variances Student’s t-test. Ntg: non-transgenic wildtype littermate control.

When we assessed behavioral performance of 3–4 months old mice on the rotarod, we surprisingly found that motor skill learning, expressed as the latency to fall, was significantly better in male hLRRK2(G2019S) mice as compared to their male wildtype littermates (**Figure 2A**). Similar but statistically insignificant trends of LRRK2-transgene dependent improved rotarod performance were observed also in female hLRRK2(G2019S) (**Figure 2A**) and male as well as female hLRRK2(WT) mice (data not shown). By the age of 10 months, the beneficial rotarod effects of high LRRK2 levels had waned (data not shown). Motility, measured as distance travelled in a homecage-like environment, was also enhanced in the first 30 min in 7 month-old but not in aged mice (**Figure 2B**). Although suggestive of some beneficial role of high LRRK2 levels on motor performance, it is not possible to correlate the transient behavioral performance changes with LRRK2 transgene expression in any particular brain area. Simply, because many areas relevant to motor behavior except cerebellum expressed the transgene (**Figure 1B,1C,S** **1,S2**). Others have reported either changes [47], [53], [54] or no changes [51], [55] in motor behaviors in mice over-expressing LRRK2(G2019S). Investigators used both different LRRK2 variants as well as transgenes with different expression profiles in heterogeneous mouse genetic backgrounds. Therefore, we believe it is not possible to draw firm conclusions unlike when comparing mouse lines made via a LRRK2 gene-specific knock-in mutagenesis approach as we demonstrated recently [30]. Furthermore, no significant changes were detected in other motor behavior-relevant tests including cocaine-induced hyperlocomotion (data not shown), behavior in the open field (**Figure S3C**), homecage running wheel performance (**Figure S3F**) or movement measured using an actimeter device (data not shown). Likewise, no changes were observed in anxiety-relevant tests in the open field, the dark/light box and the elevated plus-maze (**Figure S3D,S3E** and data not shown) or hippocampus-dependent spatial reference learning in the Morris watermaze (**Figure S3A,S3B**). Taken together, it seems clear that high levels of G2019S or wildtype LRRK2 protein are well tolerated in many forebrain, hindbrain and brainstem neurons *in vivo* with little bearing on neuronal network functions required to perform a variety of behavioral tasks. At first, this seems quite surprising. LRRK2 has been implicated in key neuronal processes such as synaptic vesicle trafficking, exo- and endocytosis [**54]**, [56], [57****], the shaping and branching of neurites [51], [58], [59], [60], [61], [62], [63], autophagy/lysosomes [30], [51], [58], [61] and neurogenesis [59]. The underlying molecular mechanisms remain to be understood but the diversity of functions suggests that LRRK2 is either involved in multiple independent signaling pathways or part of a central signaling complex with multiple in- and outputs. The difficulties thus far to discover clear LRRK2-dependent brain phenotypes in mice seems to suggest that LRRK2 roles are subject to compensation and success in unmasking these may dependent on choosing the right tasks.

Next, we analyzed whether increased levels of LRRK2 compromise neuronal integrity *in vivo* by triggering neuropathophysiological changes via endogenous aSN or Tau. Brains of aged hLRRK2(G2019S) mice (15 months) showed no differences in the levels of aSN, P-S129-aSN, Tau and P202-Tau as compared to wildtype littermate brain (**Figure 2C**). Note, P-S129-aSN levels in wildtype mouse brain are very low and variable and this pattern did not change in LRRK2 over-expressing mice. The levels of Tau and in particular P202-Tau are also variable from animal to animal. Overall, P202-Tau levels seemed slightly higher in hLRRK2(G2019S) mouse brains but robust increases as described by others [33], [54], [61], [63] were not observed and the effects we observed remained statistically insignificant. In summary, in our hands, excessive levels of wildtype or mutant LRRK2 failed to induce histopathological hallmarks of α-synucleinopathy and tauopathy in Thy1-transgene targeted mouse neurons. Altogether, these findings suggest that high LRRK2 levels do not compromise endogenous aSN and Tau homeostasis. This contrasts with findings reported by others who documented alterations in aSN and/or Tau levels following LRRK2 over-expression [33], [52], [54], [55], [61], [63], [64], [65], [66]. Perhaps cellular context is a key determining factor in this process. Findings in postmortem brains of LRRK2 mutation carriers with PD show occasionally tauopathy and much more frequently α-synucleinopathy although more precise estimates of their prevalence still await larger number of cases to be investigated [10], [35], [36], [37], [38], [39], [40], [67], [68], [69]. Whether these proteinopathies occur mainly in neuronal subtypes which orthologues in the mouse are not targeted by Thy1 transgenes remains unresolved but seems rather unlikely. In agreement with results reported by Daher et al. [46] and Lin et al. [47], also our results fail to provide clear evidence that links LRRK2 protein abundance to alterations in endogenous aSN or Tau homeostasis.

**Figure 3:**
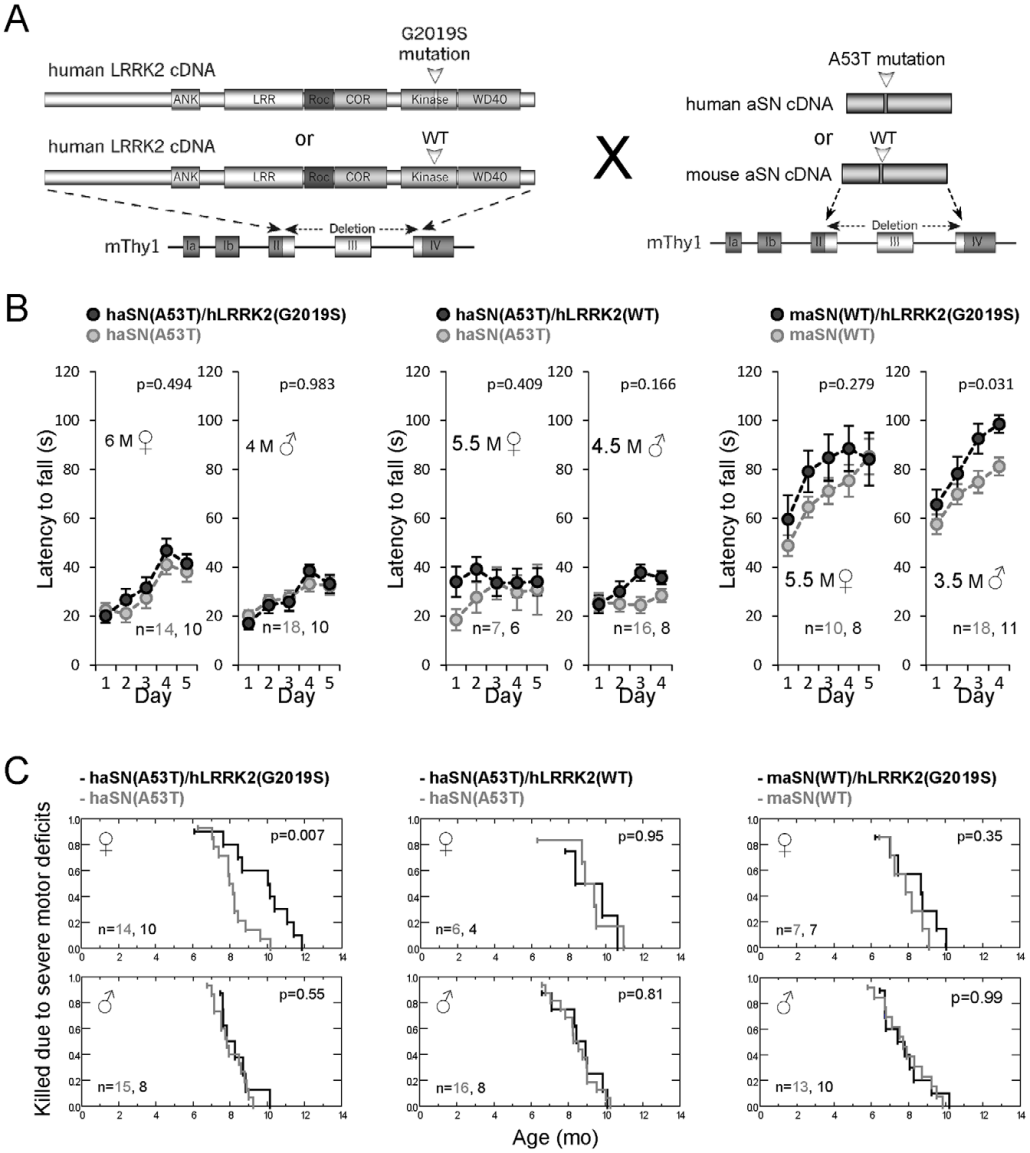
High LRRK2 transgene levels do not exacerbate α-synuclein-driven phenotypes. (A) Schematic representation of the four different transgenic lines used to generate double transgenics. (B) 3-Step accelerated rotarod performance of females and males comparing single and double transgenics. The different genotypes and the number of mice per genotype are indicated. p-values were determined by repeated measures ANOVA (group effects for the respective panels: 1: F(1,22) = 0.483, p = 0.494; 2: F(1,26) = 0.000, p = 0.983; 3: F(1,11) = 0.738, p = 0.409; 4: F(1,22) = 2.048, p = 0.166; 5: F(1,16) = 1.255, p = 0.279; 6: F(1,27) = 5.171, p = 0.031). (C) Kaplan-Meier curves showing the time-of-sacrifice when mice had to be killed because of too severe motor deficits (1 = 100% and 0 = 0% of mice alive). The different genotypes, gender, number of mice per genotype and the p-values (nonparametric Kaplan-Meier) are indicated.

It can be argued that the failure to detect such links might be due to the fact that normal endogenous aSN and Tau levels are insufficient to detect LRRK2-mediated effects on these proteins. Therefore, we tested whether high LRRK2 levels can exacerbate transgene-driven α-synucleinopathy. To this end, double-transgenic mice were generated that co-express, in a large number of neurons hLRRK2(G2019S) or hLRRK2(WT) together with high levels of the familial PD-causing aSN(A53T) mutant also under the control of the exact same Thy-1 transgene used to express the LRRK2 variants. These lines are referred to as: haSN(A53T)/hLRRK2(G2019S), haSN(A53T)/hLRRK2(WT) and maSN(WT)/hLRRK2(G2019S) (**Figure 3A**). Immunofluorescence studies revealed extensive co-localization within many neurons in different brain regions of transgene-derived LRRK2 and aSN (**Figure S4**). As reported earlier, haSN(A53T) mice developed motor coordination deficits (**Figure 3B** and [49]) earlier than maSN(WT) mice (**Figure 3B** and [48]). Co-expression of LRRK2 had no influence on the declining motor skill learning phenotype of male and female haSN(A53T) mice whereas in maSN(WT)/hLRRK2(G2019S) double transgenics, it had a small but significant benefit on motor performance that was most prominent in males (**Figure 3B**). A similar sex-dependant benefit of high LRRK2 levels was noted in single hLRRK2(G2019S) transgenics (**Figure 2A**). Therefore, LRRK2-mediated effects in the double transgenic background are likely not due to a reversal of aSN-transgene-induced motor deficits precluding us from pursuing similar assessments in maSN(WT)/hLRRK2(WT) double transgenics.

Around 6 months of age, haSN(A53T) and maSN(WT) mice began to show severe motor deficits, hypokinesia and weight loss. At this stage, animals were sacrificed in accordance with local regulations. The age-of-onset of this late-stage phenotype varied from animal to animal between 6 and 11 months with no gender effect (**Figure 3C**). High LRRK2 levels did not modify the progression and features of the late-stage phenotype with one exception: a delay in onset was observed in female haSN(A53T)/hLRRK2(G2019S) double transgenics as compared with haSN(A53T) single transgenic female littermates (**Figure 3C** **).** No significant difference in life expectancy was observed in their male counterparts and in both sexes of maSN(WT)/hLRRK2(G2019S) and haSN(A53T)/hLRRK2(WT) mice (**Figure 3C**). These results are consistent with those of Daher et al. who also reported no effect on life expectancy in their double transgenic mice [46]. The in our hands observed female sex-restricted effect specific to the haSN(A53T)/hLRRK2(G2019S) double transgenic line are most likely caused by a integration-site effect although other possibilities can not be ruled out.

**Figure 4:**
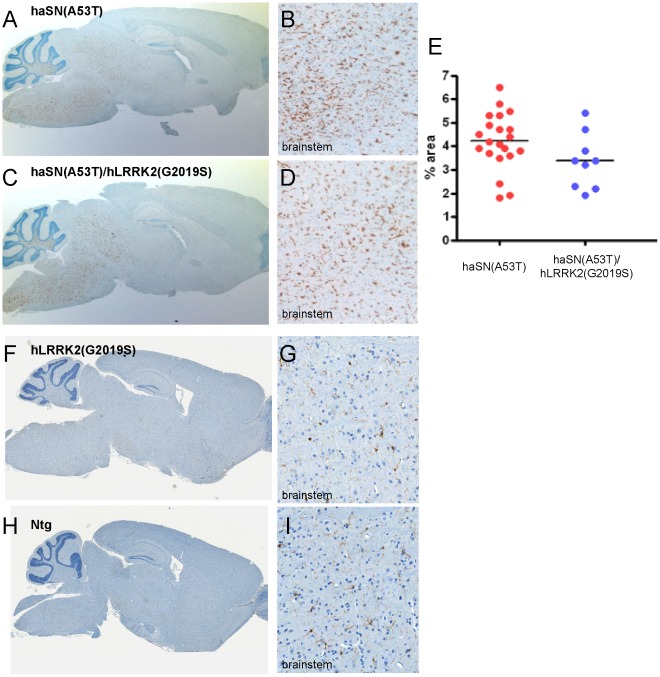
Microgliosis in end-stage haSN(A53T) transgenic mouse brain is unaltered by high LRRK2 levels. DAB-immunohistochemistry for Iba1 shows activated microglia on a representative sagittal brain section of a haSN(A53T) mouse (A and 20×higher magnification from brainstem in B) and a haSN(A53T)/hLRRK2(G2019S) double transgenic mouse (C and 20×higher magnification from brainstem in D). (E) Quantification of the brainstem results. Values represent % of the area in the brainstem that is covered by Iba1-positive microglia. p-value (p = 0.179) was determined by two-tailed, unequal variances Student’s t-test. Dots represent quantifications of single individuals. Control images obtained from a separate experiment but from littermate hLRRK2(G2019S) single transgenic (F and 20×higher magnification from brainstem in G) and from non-transgenic wildtype littermate control (Ntg) (H and 20×higher magnification from brainstem in I) mouse.

End-stage haSN(A53T) mice, as described earlier [49], and double transgenic haSN(A53T)/hLRRK2(G2019S) mice both showed a massive and widespread microgliosis (IBA1-positive microglia) in hindbrain structures (**Figure 4A–D**) as compared to control non-transgenic littermates and single transgenic hLRRK2(G2019S) mice (**Figure 4F–I**). Quantitative analysis did not reveal significant differences between genotypes (**Figure 4E**) which is in accordance with the results on gliosis in hindbrain structures published by Daher et al. [46] but contrasts with the exacerbated forebrain (mainly striatum) gliosis observed in double versus single transgenics reported by Lin et al. [47]. If anything, a non-significant trend (p = 0.179) towards reduced microgliosis was observed in double as compared to single transgenics. We had a similar finding in maSN(WT) and maSN(WT)/hLRRK2(G2019S) mice (**Figure S5**). End-stage astrogliosis as described earlier [48], [49] was also similar in maSN(WT) and haSN(A53T) mice with and without co-expressing of LRRK2 (**Figure S6** and data not shown). It seems likely, that the discrepancy between the accelerated LRRK2-dependent aSN-pathology and gliosis mainly in the striatum as reported by Lin et al. [47] contrary to its lack in other forebrain, hindbrain and brainstem regions as reported here by us and others [46] might have its cause in the much higher levels of CaMKII promoter-driven transgene expression Lin et al. achieved in the striatum [47].

**Figure 5:**
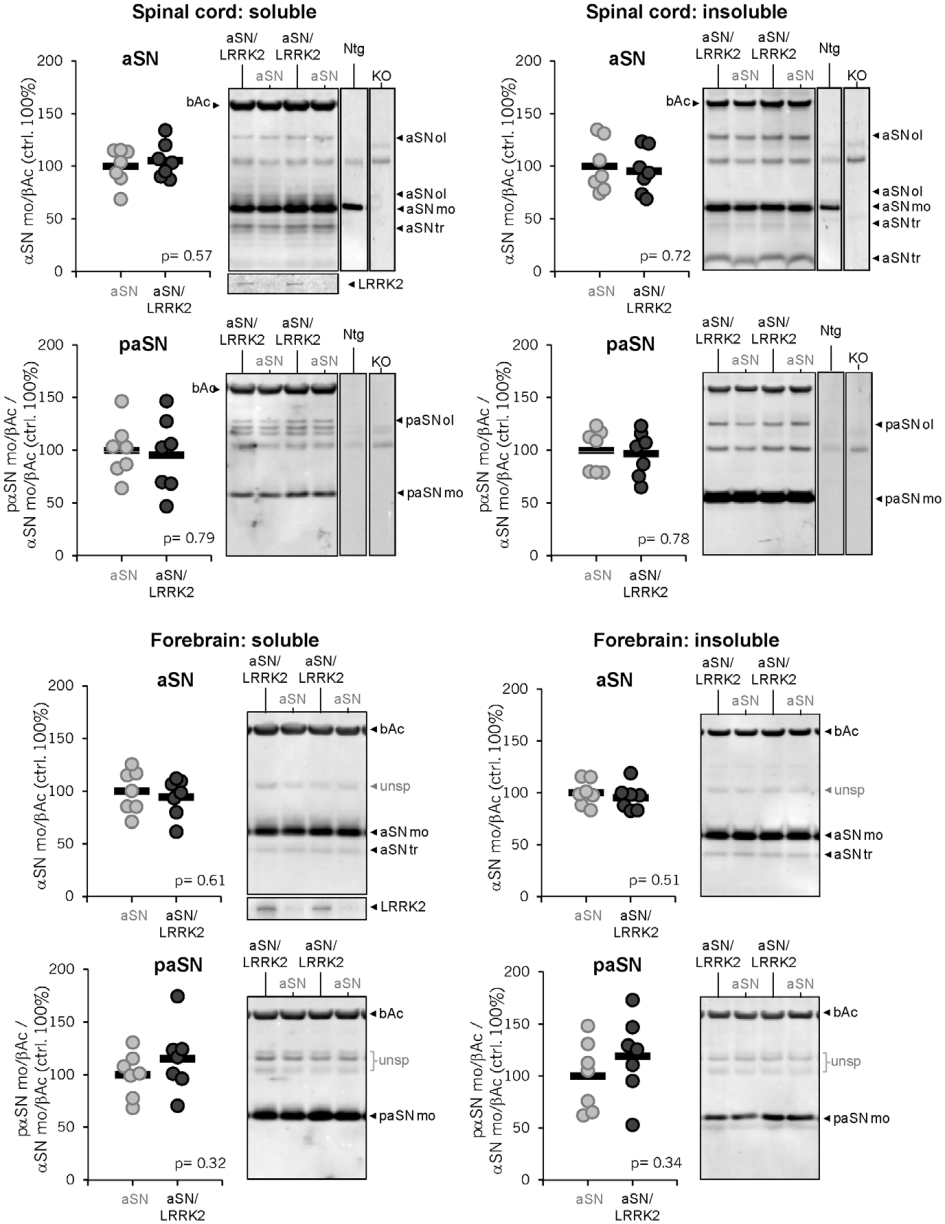
aSN and phospho-S129-aSN protein levels in spinal cord and forebrain of end-stage disease single and double transgenic mice. Tris-soluble and -insoluble fractions of spinal cord and forebrain lysates were immunoblotted and stained with antibodies detecting total α-synuclein (aSN) or specifically phosphorylated S129-aSN (paSN). β-actin (βAc) levels were measured as loading control and for normalization. For reference, LRRK2 levels detected via immunoblot are shown comparing single and double-transgenics. Different α-synuclein protein species/forms are marked as follows: mo, monomer; ol, oligomer; tr, truncated. For reference, in the upper panels the performance and specificity of the antibodies are illustrated in the two right lanes comparing WT and KO (aSN knock-out) brain samples and were added to indicate unspecific cross-reactive proteins (taken from Figure S7). Graphs represent quantifications of monomeric aSN and paSN/aSN, all normalized to βAc. Circles represent individual mice, the means are indicated as horizontal bars and % are normalized to the levels in haSN(A53T) single transgenics. p-values were determined by two-tailed, unequal variances Student’s t-test. Genotypes: aSN = haSN(A53T), aSN/LRRK2 = haSN(A53T)/hLRRK2(G2019S), Ntg = non-transgenic wildtype littermate control and KO = aSN knock-out mice.

**Figure 6:**
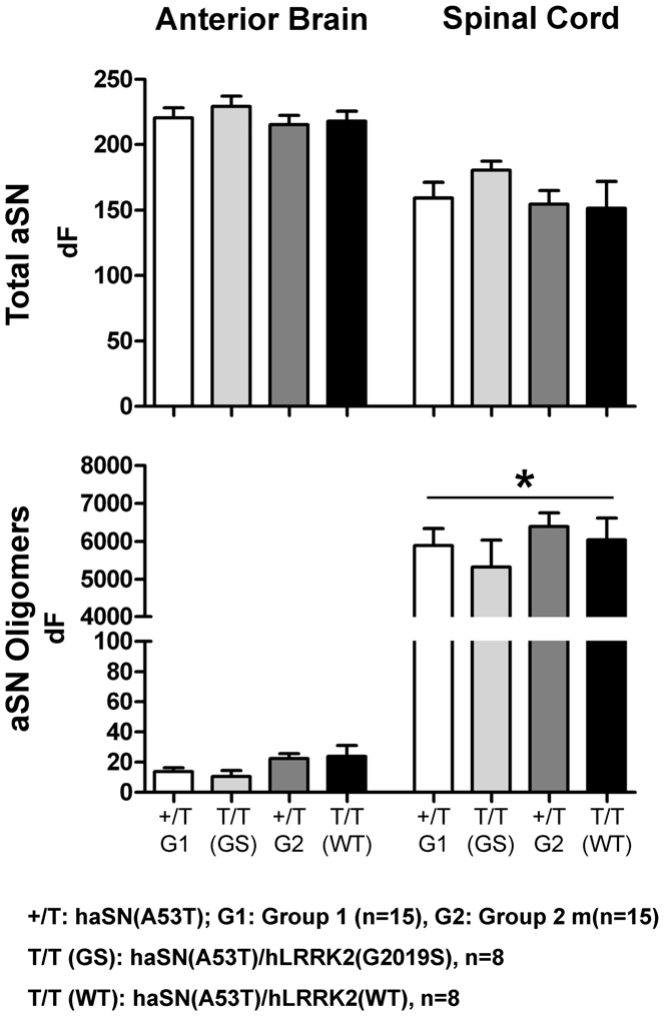
aSN oligomers, detected by TR-FRET, are dramatically increased in the spinal cord of mice with end-stage disease, relative to forebrain, independently of LRRK2(G2019S) expression. TR-FRET analysis for total or oligomeric α-synuclein (aSN) was performed in 384-well microtiter plates on homogenates of the indicated brain regions. Antibody combinations used in TR-FRET were NovSyn2-Tb/NovSyn3-d2 for total aSN, and NovSyn3-Tb/NovSyn3-d2 for oligomeric aSN. dF is the percent increase of the aSN signal above buffer background. Individual samples were measured in duplicate and the average used to calculate the plotted group means. p-values (asterisk indicate p<0.05) were determined by 1way ANOVA (Kruskal-Wallis test).

Biochemical analysis showed that end-stage haSN(A53T) but not their wildtype non-transgenic littermates had accumulated significant levels of S129-phosphorylated aSN oligomers and aSN truncated species in both the soluble and the insoluble fraction of spinal cord extracts (**Figure 5** **;** please note, non-transgenic wildtype controls (Ntg) and KO lanes were taken from a blot shown also in **Figure S7**). The forebrain was largely devoid of aSN oligomers (**Figure 5** and [50]). We further confirmed these findings by also using novel time-resolved Foerster-resonance energy transfer (TR-FRET) based assays that detect either total aSN or specifically aSN oligomers (**Figure 6** and Bidinosti et al., submitted). In the spinal cord, aSN oligomers appear in an age-dependent fashion and become prominent only close to end-stage disease when the mice also display fulminate neuronal aSN and ubiquitin histopathology [48], [50]. Most importantly, none of these aSN disease-related protein patterns on gel or *in situ* changed as a result of LRRK2 co-expression. Again, these results largely agree with the findings in hindbrain regions reported by Daher et al. [46] but they differ from the LRRK2-promoting effects on aggregation and somatic accumulation in mainly the striatum reported by Lin et al. [47]. It is however important to note that our Thy-1-based transgenes are poorly expressed in the striatum and the prion-based transgenes used by Daher et al. [46] seem more hindbrain-selective. Therefore, altogether these findings tempt us to speculate that LRRK2-mediated exacerbation of aSN neuropathology might be highly cell-type and brain-region dependent. Our data provide support for no such direct interplay in many forebrain and brainstem neurons that express Thy1-based transgenes.

Analysis on denaturing gels showed that P-129S-aSN levels in the soluble fraction are much lower in spinal cord as compared to forebrain (**Figure 5**). The opposite holds for P-129S-aSN levels in the insoluble fraction (**Figure 5**) which is not surprising and in line with our previously published immunohistological analysis showing abundant localization of P-S129-aSN in microscopically visible aggregates inside affected spinal and brainstem neurons [48]. Resolving the same soluble protein fractions on native gels showed that P-S129-aSN was present in forebrain but not in brainstem (**Figure S8**). All these analysis basically indicate that P-S129-aSN in the histopathologically affected brain areas is present mainly in insoluble aggregates which is in line with our earlier reported immunohistochemical, electron microscopy (EM) and immuno-EM analysis [48]. High LRRK2 levels in the double transgenics did not significantly change the levels and protein patterns of monomeric and P-S129- aSN, neither in the soluble nor in the insoluble fractions (**Figure 5**). End-stage haSN(A53T)/hLRRK2(G2019S) mice with ages ranging from 6–12 months also revealed no qualitative and quantitative differences in aSN protein patterns (**Figure S9**). Neither did TR-FRET analysis in endstage haSN(A53T)/hLRRK2(G2019S) and maSN(WT)/hLRRK2(G2019S) mice (**Figure 6** **,S10**). Also immunohistological analysis failed to reveal obvious differences in aSN staining patterns when comparing haSN(A53T) single and haSN(A53T)/hLRRK2(G2019S) double transgenic mice (**Figure S11**). Therefore, regardless of the variable age at which mice reached end-stage disease, α-synucleinopathy features in individual animals were essentially strikingly similar and indistinguishable between single and double transgenics. Of course, we may have missed effects that might have occurred in a specific timeframe in younger animals that predominantly contain monomeric aSN [48], [50]. A detailed analysis of time-dependent micro- and astrogliosis might reveal further insight but will be technically very challenging because of the variable age at end-stage disease. It is also difficult to exclude that the aSN transgenic models used override and therefore mask LRRK2 modifying effects on aSN. Lastly, we should not exclude that reduced rather than increased LRRK2 abundance can modify α-synucleinopathy. Recent findings in LRRK2 knockout, G2019S knock-in and kinase-dead knock-in mice suggest that LRRK2 steady-state levels can change in LRRK2-variant, tissue and brain-region dependent fashions [30]. Not reported before, we have observed a consistent reduction in LRRK2 protein levels in the striatum but not e.g. the cerebellum of LRRK2 G2019S knock-in mice (**Figure S12**). In organs like the kidney, we did see a change in the abundance of the kinase-dead LRRK2 variant but not for the G2019S variant [30]. Thus, cell-type specific differences in the regulation of LRRK2 variants and their steady-state levels might eventually turn out to be a determining factor in PD pathogenesis.

## Materials and Methods

### Ethics Statement

All experiments were carried out in accordance with the authorization guidelines of the Swiss federal and cantonal veterinary offices for the care and use of laboratory animals. Studies described in this report were approved by the Swiss cantonal veterinary office and performed according to Novartis animal license number 2063.

### Transgenic Mice

Human LRRK2 wildtype or G2019S mutated cDNA (7665 bp) was cloned into the Thy1 cassette [49] and transgenic Thy1-hLRRK2 mice were generated by pronuclear injection (C57Bl/6 mouse eggs) of linearized (NotI) and purified minigene. Transgenic mice were selected by PCR analysis of tail DNA with primers Thy1for2 (5′-GGG CTG ACC TGG ACA TTA GG-3′) and PARK8rev2 (5′-GGC GAA TTC TGC AGA TAT CC-3′), which amplified with a standard PCR protocol an 870 bp DNA fragment.

Thy1-haSN(A53T) [49] and Thy1-maSN [48] were genotyped by using primers HP45 (5′-ACA CCC CTA AAG CAT ACA GTC AGA CC-3′) and HP42 (5′-TGG GCA CAT TGG AAC TGA GCA CTT-3′), amplified DNA fragment: 1200 bp. Double transgenic mice Thy1-hLRRK2/Thy1-aSN were identified by PCR analysis of tail DNA using two independent rounds of PCR, one for Thy1-hLRRK2 and Thy1-aSN, respectively. Transgenic mice were kept in C57BL/6 background. For analyses males and females were used.

Alternatively, single and double transgenic mice were selected by standard Taqman PCR analysis of tail or ear DNA with primers and fluorescently labeled probes: Thy1for2 (5′-GGG CTG ACC TGG ACA TTA GG-3′), Thy1rev2 (5′-GGT CTG ACT GTA TGC TTT AGG G-3′) and Thy1-probe (5′-FAM-CCA GAG ACT GGC TAC ACA GCG ATA TGA C-TAMRA-3′). As endogenous control PCR: α-synuclein (αSN-5′ (5′-GCT GGA AAG ACA AAA GAG GG-3′), αSN-3′ (5′-ATT CTC TCA CCT CCA CAC AG-3′), αSN-probe (5′-FAM or YYE-TGG CTG GTG TGT GGT GTC TGA TT-TAMRA-3′)) or LRRK2 (LRRK2.for (5′-TGT ATC CCA ATG CTG CCA TC-3′), LRRK2.rev (5′-CTA TAT CTC CTA GAC CCA CAC-3′), LRRK2.Ex41.probe (5′-YYE-TGG GAA TAA AGA CAT CAG AGG GCA C-TAMRA-3′)). Rotorgene 3000 was used for PCR reaction (95°C 10 min, 40 cycles of 95°C 15 sec and 60°C 1 min).

### Immunoblot Analysis

All work was carried out at 4°C. Mouse brain was added to 5 ml homogenization buffer (20 mM Tris-HCl, pH 7.4, 0.25 M sucrose, 1 mM EDTA, 1 mM EGTA, 0.5 mM PMSF, 5 µg/ml pepstatin A and leupeptin, okadaic acid 0.01 µM, calyculin 0.1 µg/ml, 1×phosphatase inhibitor cocktail (PIERCE)) without any detergent, then homogenized (Precellys24, Bertin Technologies), incubated on ice for 30 min and then centrifuged at 13000 rpm for 20 minutes at 4°C. The protein concentration of the supernatant was determined using a BCA protein assay kit (Bio-Rad Laboratories). For immunoblot analysis, 10 µg/slot of supernatant (treated with NuPAGE LDS sample buffer (4×) and NuPAGE sample reducing agent (10×) at 95°C for 5 min) was analyzed by PAGE (NuPAGE Novex Bis-Tris gels (4–12%), Tris-Acetate (3–8%), Invitrogen). The pellet fraction was dissolved in the same homogenization buffer as above without detergent (10×volume of the tissue weight) and then treated like and used according to supernatant samples. For blotting XCell II blot module (Invitrogen) was used. Detection was performed using the following antibodies: Mouse monoclonal antibodies α-synuclein (1∶5000, BD Biosciences), phosphorylated α-synuclein (1∶5000, WAKO) ); rabbit anti-LRRK2 home-made (1∶500); mouse anti-Tau (1∶2000, TAU-5, Biosource); mouse anti-P202-Tau (1∶500, AT8, MN1020, Pierce); rabbit anti-TH (1∶5000, Chemicon AB152); rabbit anti-DARPP32 (1∶5000, Chemicon AB1656); rabbit anti-GAD (1∶5000, Chemicon AB1511). Membranes were washed 4 times for 5 min at room temperature in PBS containing 0.05% or 0.1% Tween20 and then incubated for 45 min (light protected) with secondary antibodies (Alexa Fluor 680, F(ab’)2 fragment of goat anti-mouse (Invitrogen); IRDye 800 CW anti-rabbit IgG (Li-cor); both 1∶5000) in Odyssey Blocking Buffer (diluted 1∶1 in PBS, containing 0.1% or 0.05% Tween20). Membranes were again washed 4× for 5 min at room temperature in PBS containing 0.05% or 0.1% Tween20, then washed 2× for 5 min at room temperature in PBS only and finally scanned on the Odyssey Li-cor System. Fluorescence intensities quantification was performed by standardization to β-actin levels using a mouse monoclonal anti β-actin clone AC-15 antibody (1∶50000, Sigma Aldrich).

### *In situ* Hybridisation

20 µm sagittal sections were prepared from fresh frozen mouse brains, fixed for 1 h in PBS buffered 4% paraformaldehyde and subjected to an automated in situ hybridisation procedure using the Ventana/Roche DiscoveryXT technology. Biefly, slides were postfixed for 4 minutes with VENTANA RiboPreb™ solution and conditioned by heat pre-treatment (12 min at 98°C in RiboCC) followed by mild proteolysis (4 min at 37°C with VENTANA protease 3). Sections were then hybridized for 6 h at 65°C with 0.1 µg/ml digoxigenin-labelled anti-sense riboprobe (corresponding to nucleotides 3537 to 4024 of the human LRRK2 gene sequence) diluted in a hybridisation solution containing one part VENTANA RiboHyb™ and two parts 2×SSC, followed by high stringency washes with 2×SSC at 75°C for 3×8 min, and post-fixation for 8 min in VENTANA RiboFix™. To visualize hybridisation signals, sections were incubated for 28 min with alkaline phosphatase labelled sheep anti-digoxygenin Fab fragments (Roche Diagnostics) diluted 1∶500 in VENTANA Discovery antibody diluent, and subjected to an alkaline phosphatase-catalyzed colour reaction with NBT/BCIP (VENTANA BlueMap™ kit) for 9 h.

### Immunohistochemistry

Antibodies used for immunohistochemistry and immunofluorescence staining: Primary antibodies: Rabbit anti-alpha synuclein (Chemicon AB5038; 1∶1000), Mouse anti-alpha synuclein (abcam 4B12; 1∶100), monoclonal mouse anti-alpha synuclein (non-commercial antibody made on behalf of Novartis); monoclonal rabbit anti-LRRK2 (Epitomics c41-2 #3514-1); Rabbit anti-GFAP (DAKO Z0334; 1∶5000), Rabbit anti-ubiquitin (DAKO Z0458; 1∶200), Rabbit anti-Iba1 (WAKO Chemicals **#019-19741; 1∶000);** Secondary antibodies: Biotinylated goat anti-mouse (Jackson ImmunoResearch; 1∶1000), Biotinylated goat anti-rabbit (Jackson ImmunoResearch; 1∶1000); Alexa488-labeled anti-rabbit IgG (Invitrogen); Alexa594-labeled anti-mouse IgG (Invitrogen).

Manual immunohistochemistry: Paraffin sections (4 µm thick) of immersion fixed mouse brains were dewaxed in xylene, rehydrated in decreasing ethanol solutions, rinsed in double-distilled water, rinsed in PBS (Phosphate-buffered saline, pH 7.4), subjected to antigen unmasking (only for GFAP and ubiquitin) and incubated for 1 h in blocking solution (from PerkinElmer Kit NEL-741B). Sections were then incubated with primary antibody (diluted in blocking solution) over night at 4°C. Slides were rinsed 4×3 min in PBS, incubated for 60 min at room temperature with biotinylated secondary antibody diluted in blocking solution, rinsed 4×3 min in PBS, incubated for 30 minutes ABC reagent (PerkinElmer TSA plus Kit) and rinsed 2×5 min in PBS. Staining was performed with AEC kit (Zymed ZUC054) according to the instructions of the supplier. Sections were counterstained for 2 min with Mayer’s Haematoxylin, immersed in ammonia water for 10 sec, rinsed with running tap water for 5 min, dehydrated in increasing ethanol solutions and xylene and mounted with Eukitt.

Manual immunofluorescence double staining to investigate co-localisation of LRRK2 and α-synuclein: 4 µm sagittal paraffin sections were de-waxed, rinsed 3× for 5 min in PBS, subjected to antigen unmasking by microwaving, incubated for 1 h with PBS containing 2% goat serum, then over night at 4°C with a mixture of mouse anti-α-synuclein (Novartis; 1∶10000) and rabbit anti-LRRK2 (MJFF2 c41, Epitomics; 1∶200) diluted in PBS/2% goat serum, washed 3× in PBS, stained with a mixture of Alexa488-labeled goat anti-rabbit and Alexa594-labeled goat anti-mouse each diluted 1∶500 in PBS, and mounted with with Prolong Gold containing DAPI nucleic acid counterstain (Invitrogen).

#### Antigen unmasking procedures to enhance staining of LRRK2 and GFAP

Following de-paraffinization and rehydration, slides were microwaved for 10 min at 90°C (T/T MEGA Milestone) in 0.1 M sodium citrate buffer pH 5.8, rinsed in PBS and transferred into blocking solution. The following steps of the immunostaining procedure were performed as described above.

#### Limited protease digestion (for ubiquitin immunostaining)

Following deparaffinization and rehydration slides were incubated for 15 min at 37°C in 0.05% pronase in 0.5 M Tis/HCl pH 7.6. Slides were then rinsed in PBS and transferred into blocking solution. The following steps of the immunostaining procedure were performed as described above.

#### Automated immunostaining of Iba1

4 µm para-sagittal paraffin sections of immersion fixed mouse brains were mounted on SuperFrost+ slides and subjected to an automated immunostaining procedure using the Discovery XT technology (Ventana/Roche diagnostics). Briefly, sections were de-paraffinized, rehydrated, subjected to antigen retrieval by heating with CC1 cell conditioning buffer (Ventana/Roche Diagnostics), incubated for 60 min at room temperature with primary antibody diluted in antibody diluent (Ventana/Roche Diagnostics), incubated with biotinylated goat anti-rabbit secondary antibody diluted in Ventana antibody dilution, reacted with DABMab kit (Ventana/Roche Diagnistics) and counterstained with blueing reagent (Ventana/Roche Diagnostics).

Microglia and astroglia were quantified by using systematic-random series of brain sections at three different anatomical planes per animal which were analyzed by a MCID image analyzer (Imaging Research, Brock University, Ontario, Canada, Program Version M7 elite). The microscopic image was digitized by use of a Roper black and white CCD TV camera and stored with 1124×1124 pixel resolution at 256 gray levels. The pixel size was calibrated using an object micrometer at 5×magnification (Leica Neoplan Objective). Using a motor driven microscope stage for exact positioning of adjacent object fields the entire brainstem of each section was analyzed. For each object field the anatomical area was defined by manual outline. For each individual section the sample area was defined by manual threshold setting (grey level). Isolated tissue artifacts were excluded by manual outline. Data were analyzed as individual counts (microglia or astroglia) to the area in %.

### TR-FRET Immunoassay Analysis

Antibodies recognizing C-terminal α-synuclein epitopes were raised in-house and are denoted as NovSyn2 and NovSyn3. Antibodies were chemically coupled with either Lumi4®-Tb (Terbium cryptate) donor fluorophore or a second generation d2 acceptor fluorophore (CisBio Bioassays). The development and characterization total or oligomeric α-synuclein TR-FRET assays is described elsewhere (Bidinosti et al., unpublished) and relies on the FRET-dependent signal generated upon coincident binding of two fluorophore-conjugated antibodies to a single α-synculein molecule or oligomer. The antibody combination of NovSyn2-Tb/NovSyn3-d2 binds two unique epitopes potentially accessible on all α-synuclein species. NovSyn3-Tb/NovSyn3-d2 however is specific for oligomeric α-synuclein via binding to multiple copies of a single epitope which are uniquely present in oligomeric species. For conducting the assay, brain regions were homogenized in 10 volumes (w/v) of lysis buffer (PBS containing 1% Triton X-100 and Complete Protease Inhibitor cocktail (Roche)). Total protein content was determined by BCA assay (Pierce) and 5 µl/well of total protein-normalized homogenates were loaded onto 384-well low-volume polystyrene microtiter plates in technical duplicates. Detection antibodies were diluted in analysis buffer (50 mM NaHPO_4_, 400 mM NaF, 0.1% BSA, and 0.05% Tween-20) and 1 µl of this solution was added such that each well contained 0.3 ng of donor fluorophore-conjugated antibody and 3 ng of acceptor fluorophore-conjugated antibody. The plates were then incubated overnight at 4°C. Sample fluorescence was measured with an EnVision Multilabel Reader (PerkinElmer) and FRET-dependent acceptor fluorescence (665 nm) was normalized to FRET-independent donor fluorescence (620 nm) for each sample. The relative total or oligomeric α-synculein content is reported as the percent increase of each sample 665 nm/620 nm ratio over that of buffer alone (dF).

### Behavior

#### Rotarod

To measure motor coordination mice were placed on a computerized treadmill (TSE rotarod system). The 3-step rotarod program consists of a modified rotarod program of three different running speeds (12 rpm, 24 rpm and 36 rpm) each for 30 sec with intervals of acceleration lasting for 10 sec. Starting speed is 4 rpm. Rotarod performance was assessed by evaluating the two best trials out of three performed in one day.

#### Grip strength

To measure forelimb grip strength, mice are allowed to grasp a handle connected to a force-measuring device (San Diego Instruments) and then pulled back with their tails until they release the handle. The best out of four consecutive trials is evaluated.

#### Open field

To measure exploratory behavior (pattern and activity), mice were placed in an open field box (70 cm×70 cm, height of walls: 30 cm) subdivided into nine quadrants with one middle quandrant. The horizontal distance travelled during 5 min was recorded by an EthoVision 3.0 system (Noldus).

#### Dark/light box

The dark/light box consists of a dark and a bright compartment. Mice were placed in the bright compartment and given the opportunity to move to the dark box for 5 min. Parameters measured by EthoVision 3.0 (Noldus) were the time spent in the bright compartment and the latency of first entry to the dark compartment. The number of transitions and the latency of first exit back to the bright compartment were measured visually.

#### Elevated plus-maze

The elevated plus-maze (80 cm from the floor) consists of four arms (length: 27 cm) arranged in right angles to each other. Two opposite arms have walls (height: 15 cm) and the two others are open. Mice are placed in the middle and are allowed to move freely for 5 min. The time spent in the open arms is recorded by EthoVision 3.0 (Noldus) and the number of entries to open arms by visual inspection.

#### Motility cages

Locomotor activity was measured with the TSE system (process control type 302013-CD, software: Motilitätsmesssystem 4.2) in motility cages (Makrolon Typ III with a lid and without embedding). Cages were changed after each animal.

#### Running wheels

Running wheels (med Associates) were placed to the homecage (Makrolon Typ III, 22×37×15 cm, with Filtertop) and the mice were allowed to freely access the wheels for 24 h for 10 days (averaged data over 24 h, software: Wheel Analysis SOF-861, environmental sensor included, med Associates).

#### Morris water maze

Mice were placed in a pool (white plastic, Ø 149 cm) filled with 24°C warm water (clouded with 2 l of skimmed milk 40 min before the start of the experiment) and was allowed for 2 min to find the escape platform (Ø 8 cm) that was localized 16 cm to the pools′ edge and 22 cm to poolś bottom, 5 mm below waterline always at at the NO quadrant. Cues were available at the walls. A session consisted of 4 trials (each max 2 min, 15 min between trials) from positions N, S, E, W, each day the start position was shifted. The room lights were switched off and indirect light was turned on 30 min before the start of the trials. For the probetrial the platform was removed and the mice were placed SW into the pool (one trial only) for 2 min. For recording and analysis Noldus EthoVision 3.0 was used.

### Animal Maintenance

The animals were housed in a temperature-controlled room that was maintained on a 12 h light/dark cycle. Food and water were available ad libitum.

### Statistics

Data are expressed as average ± SEM. Rotarod and horizontal locomoter activity curves were analyzed by repeated measure ANOVA. The survival curves were analyzed by nonparametric Kaplan-Meier test. TR-FRET was analyzed by 1way ANOVA (Kruskal-Wallis test). Statistical analyses of the other graphs were performed using two-tailed, unequal variances Students t-test.

## Supporting Information

Figure S1 **LRRK2 protein levels in hLRRK2(G2019S) and hLRRK2(WT) mice.** Immunoblots of protein extracts from different brain regions of non-transgenic wildtype littermate control (Ntg), hLRRK2(G2019S) and hLRRK2(WT) mice (5–7 months old) detecting LRRK2. β-actin served as loading control. Ntg and LRRK2 knock-out (KO) served as controls to indicate LRRK2 antibody specificity for the cerebellum immunoblot (experiment performed separately). Note that the abundance of some unspecific but LRRK2 antibody cross-reacting proteins seems slightly increased. We don’t know the reason for this and the identity of these proteins remains unknown. Nonetheless, the Western analysis of cerebellar extracts comparing LRRK2 KO versus non-transgenic tissue clearly shows that these proteins are unrelated to LRRK2.
(TIF)

Figure S2 **hLRRK2(G2019S) and hLRRK2(WT) transgene mRNA expression in the mouse brain.** Transgene hLRRK2(G2019S) and hLRRK2(WT) mRNA expression pattern comparing transgenic and non-transgenic wildtype littermate control (Ntg) mouse brain regions visualized using a DIG-labeled cDNA probe. Note the weak expression of hLRRK2(WT) transgene in striatum that was confirmed in immunoblot analysis.
(TIF)

Figure S3 **Behavioral characterization of the hLRRK2(G2019S) mouse line.** Performance is shown of non-transgenic wildtype littermate control (Ntg) and transgenic hLRRK2(G2019S) male mice in (A) the water maze learning task (one session per day consisted of four trials) at 3.5 months of age; (B) Probetrial of the water maze. Platform is located in the north-east (NE) quadrant (NW: north-west; SE: south-east; SW: south-west); (C) open field behavior expressed as total distance moved (age of the animals: 2 months); (D) dark/light box behavior expressed as the latency before entering the dark compartment (age of the animals: 2 months); (E) the elevated plus-maze task with results expressed as time spent in the open arms (age of the animals: 2 months) and (F) on the running wheel shown as activity over time during 24 hrs in the homecage (age of the animals: 23 months). Genotypes and n values are indicated.
(TIF)

Figure S4 **Co-localization of LRRK2 and aSN in haSN(WT)/hLRRK2(G2019S) mouse brain.** Immunofluorescence for aSN (red) and LRRK2 (green) of sagittal brain section of Ntg and haSN(A53T)/hLRRK2(G2019S) mice for (A) cortex (20×magnification) and (B) brainstem (40×magnification). Co-localization of aSN and LRRK2 (overlay of red and green) is depicted by white arrows; gray arrows point to aSN-positive, LRRK2-negative cells.
(TIF)

Figure S5 **Microgliosis in maSN(WT) and maSN(WT)/hLRRK2(G2019S) end-stage mouse brain.** DAB-immunohistochemistry for Iba1 shows activated microglia on a representative sagittal brain section of a maSN(WT) single (A and 20×magnification of brainstem in B) and a haSN(A53T)/hLRRK2(G2019S) double transgenic mouse (C and 20×magnification of brainstem in D). (E) Quantification of the area in the brainstem that is covered by Iba1-positive microglia plotted as % of total area. Dots represent quantifications of individual mice. Control images are shown in Figure 4.
(TIF)

Figure S6 **High neuronal levels of LRRK2 do not worsen astrocytosis in haSN(A53T) end-stage mouse brain.** DAB-immunohistochemistry for GFAP shows astrocytosis on a represetative sagittal brain section of (A) maSN(WT), (B) haSN(A53T), (C) haSN(A53T)/hLRRK2(G2019S) (20×magnification from brainstem is shown in (D)), (E) maSN(WT)/hLRRK2(G2019S), (F) hLRRK2(G2019S) single transgenic and (G) non-transgenic wildtype littermate control (Ntg) mice. Please note, experiments for (E–G) were performed separately.
(TIF)

Figure S7 **Specificity of aSN antibodies used.** Immunoblots of spinal cord extracts from non-transgenic wildtype littermate control (Ntg), haSN(A53T) and aSN knock-out (KO) mice detecting total α-synuclein (aSN), phosphorylated S129-aSN (P-S129-aSN), and unspecific protein species cross-reacting with each antibody (aSN KO lanes). Parts of these results are shown also in Figure 5 for illustration purposes.
(TIF)

Figure S8 **aSN protein species resolved on native gels.** Immunoblotting results are shown using antibodies detecting total α-synuclein (aSN) and phosphorylated S129-aSN (P-S129-aSN) (arrows) in soluble protein extracts of forebrain and brainstem comparing non-transgenic wildtype littermate control (Ntg) and haSN(A53T) mice. unsp.: refers to non-aSN proteins cross-reacting with antibody.
(TIF)

Figure S9 **aSN and phospho-S129-aSN levels in spinal cord at different ages of end-stage haSN(A53T)/hLRRK2(G2019S).** Immunoblotting of Tris-soluble (supernatant) and Tris-insoluble (pellet) fractions of spinal cord lysates. Blots were stained with antibodies against α-synuclein (αSN) or phosphorylated S129-aSN (pαSN) and β-actin (βactin). The different aSN species are indicated: mono, mo; monomer, olig; oligomer; trunc, truncated. Age indicates the time when each mouse had reached the stage of illness that required us to kill the animal.
(TIF)

Figure S10 **aSN oligomers, detected by TR-FRET, are increased in the spinal cord of end-stage maSN(WT) mice, independently of hLRRK2(G2019S) co-expression.** TR-FRET analysis was performed as in Figure 6 on homogenates of the indicated brain regions. Each sample was measured in duplicates. p-values (asterisks indicate p<0.01) were determined by 1way ANOVA (Kruskal-Wallis test).
(TIF)

Figure S11 **aSN brain histopathology in end-stage haSN(A53T) and** **haSN(A53T)/hLRRK2(G2019S) mice.** For each genotype, a representative section (10×magnification) stained against aSN is shown of the brainstem (reticular formation), midbrain (deep mesencephalic nucleus), hippocampus and cortex (primary motor cortex) of a haSN(A53T) and a haSN(A53T)/hLRRK2(G2019S) mouse.
(TIF)

Figure S12 **Reduced LRRK2 protein levels in LRRK2(G2019S) knock-in mouse striatum.** (A) Immunoblotting results of striatal lysates of Ntg and KI (LRRK2(G2019S) knock-in) female mice are shown (age: 7.5 months). Protein levels determined included LRRK2, DARPP-32 and TH. β-actin (βAc) was used as loading control and for normalization. Circles represent individual mice; the means are indicated as horizontal bars and % are normalized to the protein levels in Ntg. (B) Immunoblotting results for LRRK2 and GAD65/67 of cerebellar lysates from Ntg and KI (LRRK2(G2019S) knock-in) female mice (age: 7.5 months) and quantification of the results. β-actin (βAc) was used as loading control and for normalization. Circles represent individual mice, the means are indicated as horizontal bars and % are normalized to levels in Ntg mice. (C) Quantification of LRRK2 immunoblot results comparing levels in Ntg and KI (LRRK2(G2019S) knock-in) lysates of dorsal, ventral (males, 6.5 months old) and total (dorsal + ventral; females, 5.5 months old) striatum. Values are expressed as % of level in Ntg. Bars indicates SEM. LRRK2 levels were normalized to β-actin and DARPP-32 (which is specifically expressed in the LRRK2-expressing GABAergic projections neurons of the striatum). The number of mice per group is indicated. p-values were determined by two-tailed, unequal variances Student’s t-test. Ntg: non-transgenic wildtype littermate control.
(TIF)
