# Supplementary figures and images for: High LRRK2 Levels Fail to Induce or Exacerbate Neuronal Alpha-Synucleinopathy in Mouse Brain
Source: PLoS One. 2012 May 15;7(5):e36581. doi: 10.1371/journal.pone.0036581 (PMC3352901; doi:10.1371/journal.pone.0036581)

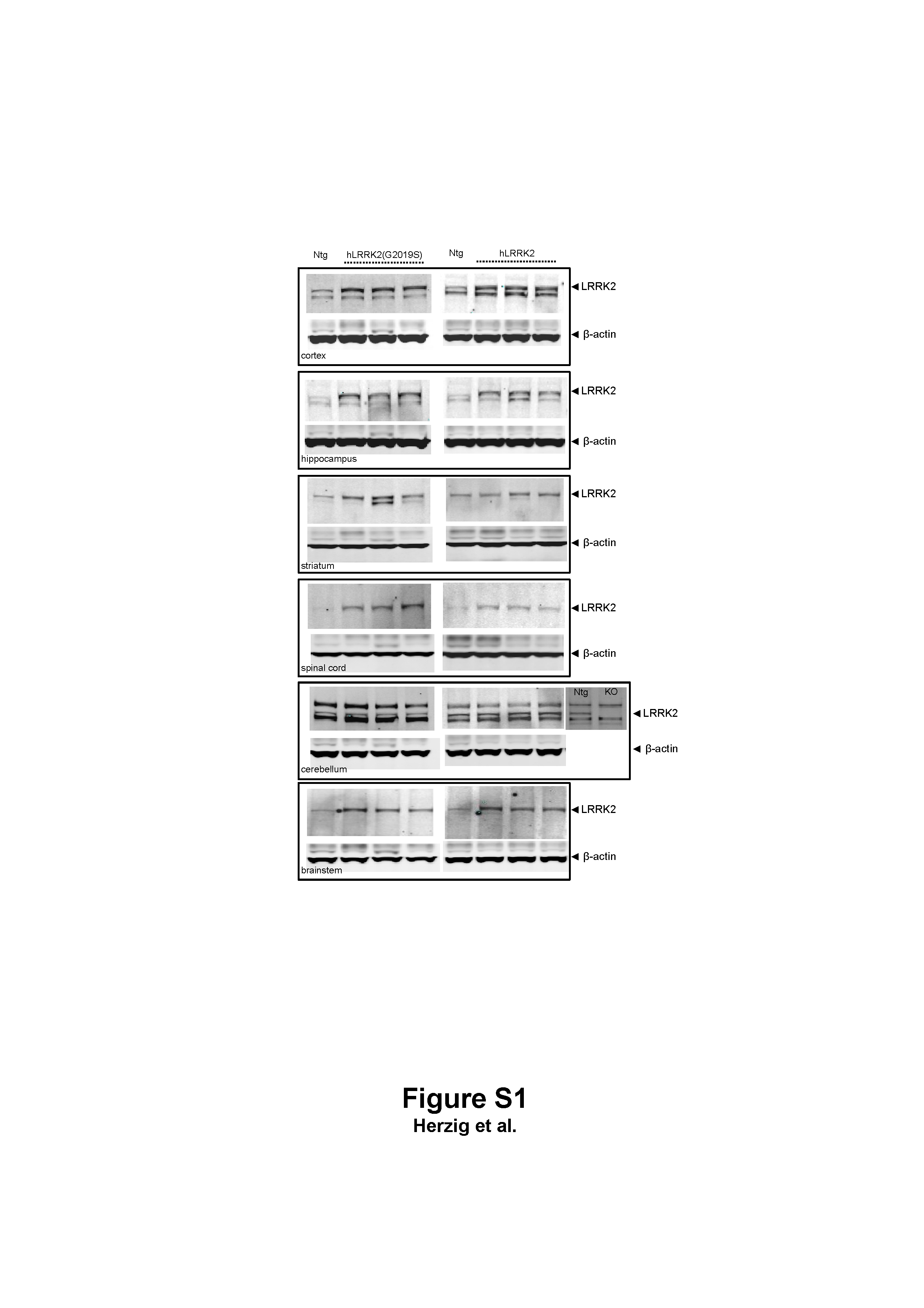

Supplement: Figure S1 — LRRK2 protein levels in hLRRK2(G2019S) and hLRRK2(WT) mice. Immunoblots of protein extracts from different brain regions of non-transgenic wildtype littermate control (Ntg), hLRRK2(G2019S) and hLRRK2(WT) mice (5–7 months old) detecting LRRK2. β-actin served as loading control. Ntg and LRRK2 knock-out (KO) served as controls to indicate LRRK2 antibody specificity for the cerebellum immunoblot (experiment performed separately). Note that the abundance of some unspecific but LRRK2 antibody cross-reacting proteins seems slightly increased. We don’t know the reason for this and the identity of these proteins remains unknown. Nonetheless, the Western analysis of cerebellar extracts comparing LRRK2 KO versus non-transgenic tissue clearly shows that these proteins are unrelated to LRRK2. (TIF) [file pone.0036581.s001.tif]

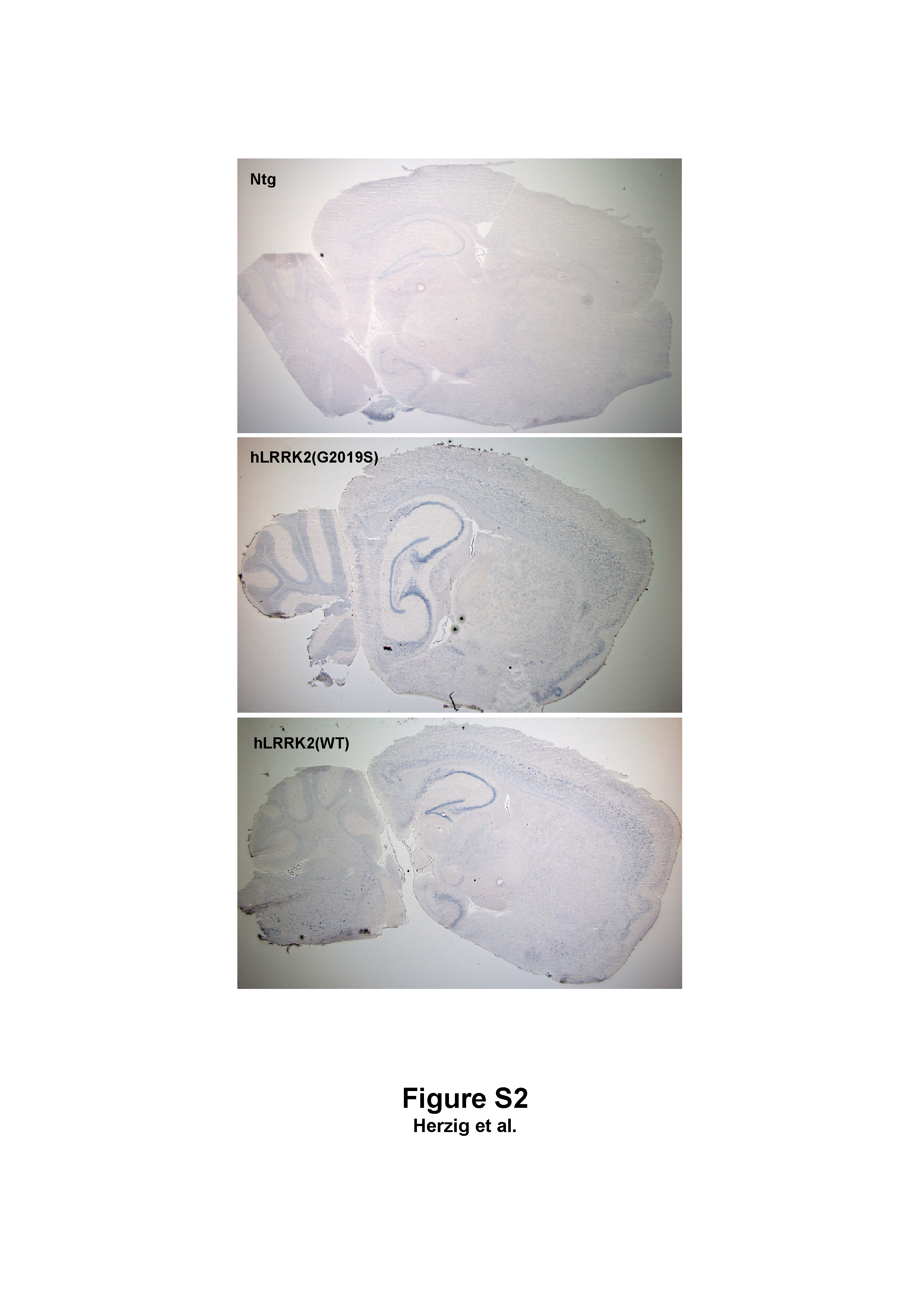

Supplement: Figure S2 — hLRRK2(G2019S) and hLRRK2(WT) transgene mRNA expression in the mouse brain. Transgene hLRRK2(G2019S) and hLRRK2(WT) mRNA expression pattern comparing transgenic and non-transgenic wildtype littermate control (Ntg) mouse brain regions visualized using a DIG-labeled cDNA probe. Note the weak expression of hLRRK2(WT) transgene in striatum that was confirmed in immunoblot analysis. (TIF) [file pone.0036581.s002.tif]

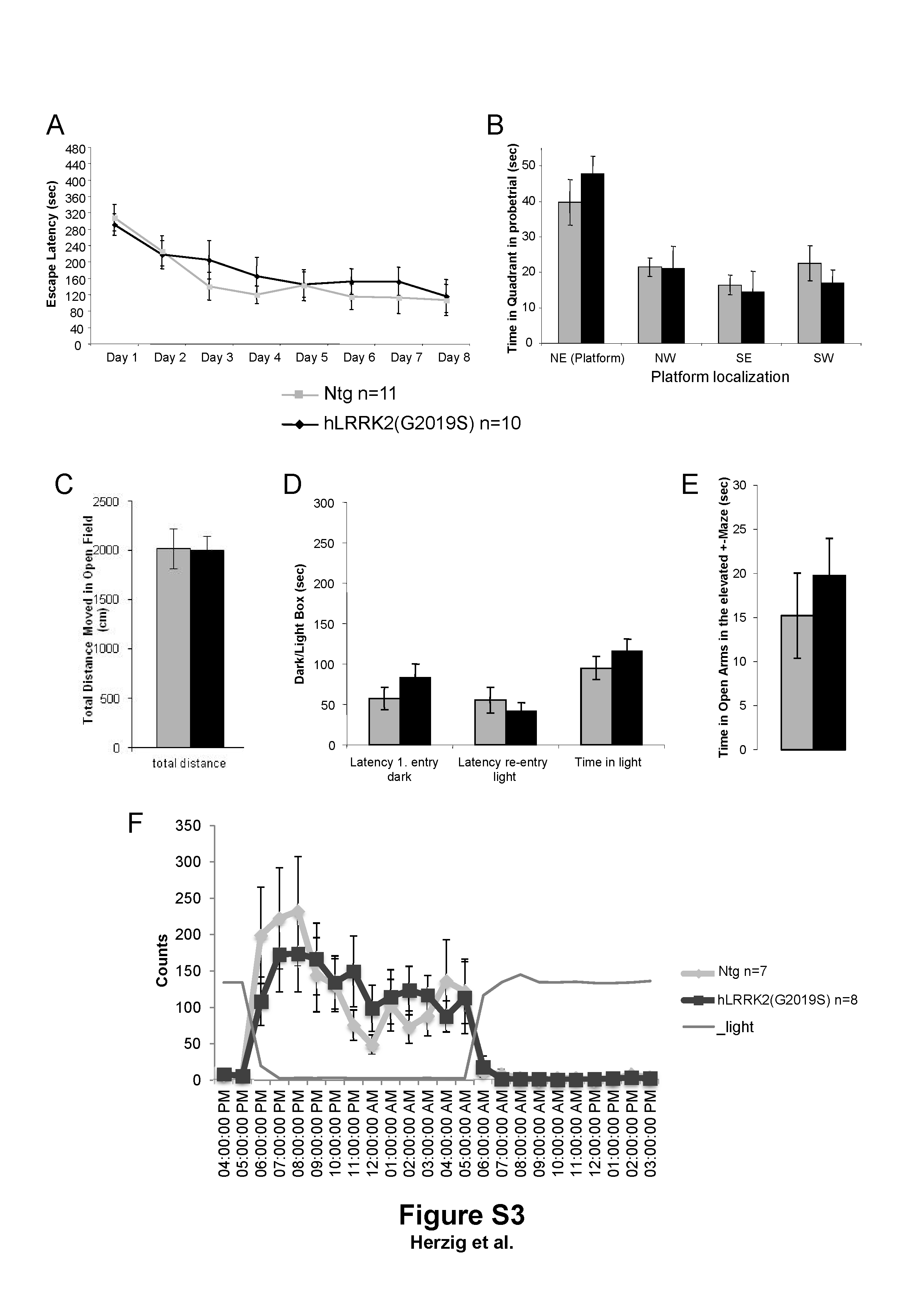

Supplement: Figure S3 — Behavioral characterization of the hLRRK2(G2019S) mouse line. Performance is shown of non-transgenic wildtype littermate control (Ntg) and transgenic hLRRK2(G2019S) male mice in (A) the water maze learning task (one session per day consisted of four trials) at 3.5 months of age; (B) Probetrial of the water maze. Platform is located in the north-east (NE) quadrant (NW: north-west; SE: south-east; SW: south-west); (C) open field behavior expressed as total distance moved (age of the animals: 2 months); (D) dark/light box behavior expressed as the latency before entering the dark compartment (age of the animals: 2 months); (E) the elevated plus-maze task with results expressed as time spent in the open arms (age of the animals: 2 months) and (F) on the running wheel shown as activity over time during 24 hrs in the homecage (age of the animals: 23 months). Genotypes and n values are indicated. (TIF) [file pone.0036581.s003.tif]

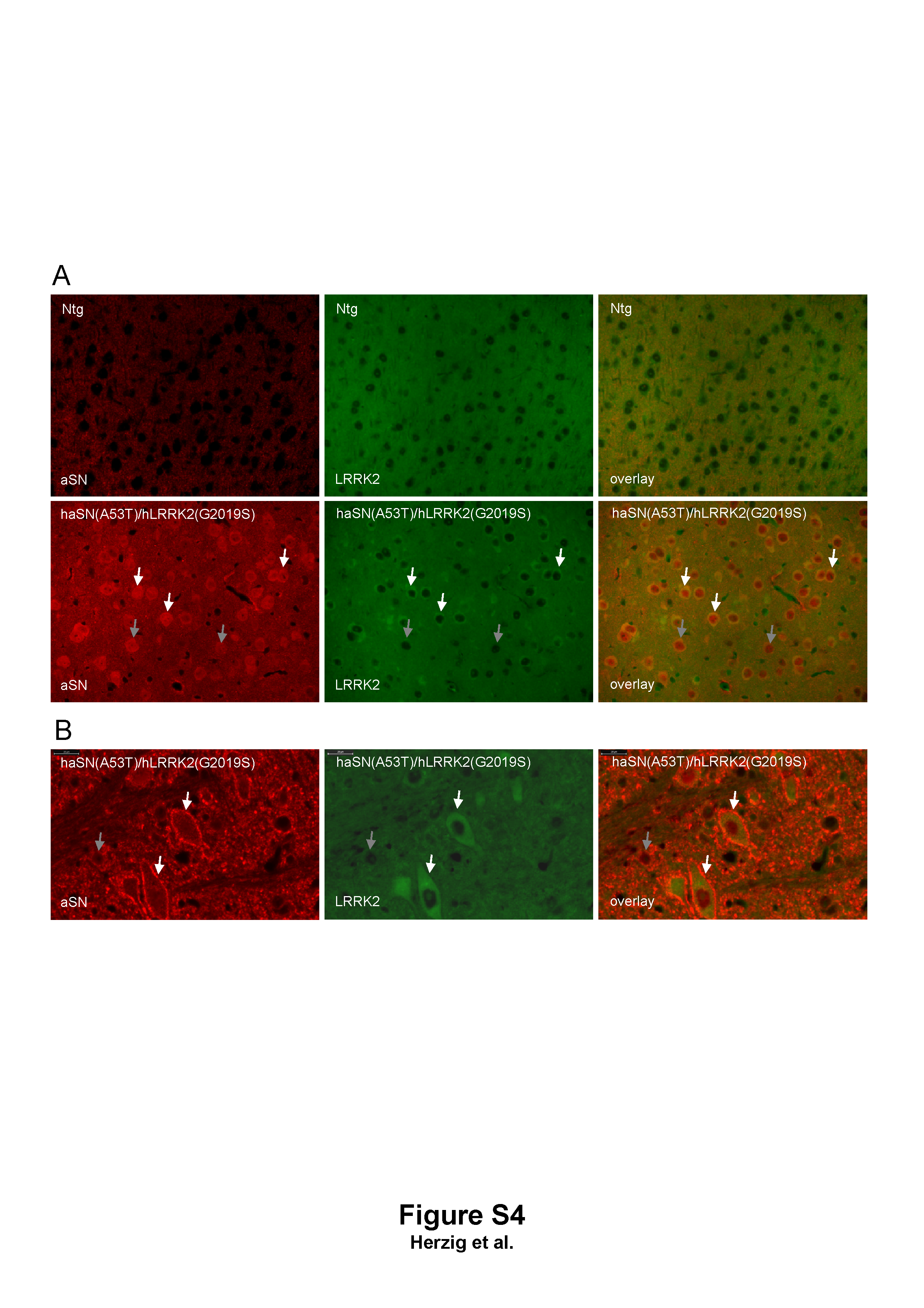

Supplement: Figure S4 — Co-localization of LRRK2 and aSN in haSN(WT)/hLRRK2(G2019S) mouse brain. Immunofluorescence for aSN (red) and LRRK2 (green) of sagittal brain section of Ntg and haSN(A53T)/hLRRK2(G2019S) mice for (A) cortex (20×magnification) and (B) brainstem (40×magnification). Co-localization of aSN and LRRK2 (overlay of red and green) is depicted by white arrows; gray arrows point to aSN-positive, LRRK2-negative cells. (TIF) [file pone.0036581.s004.tif]

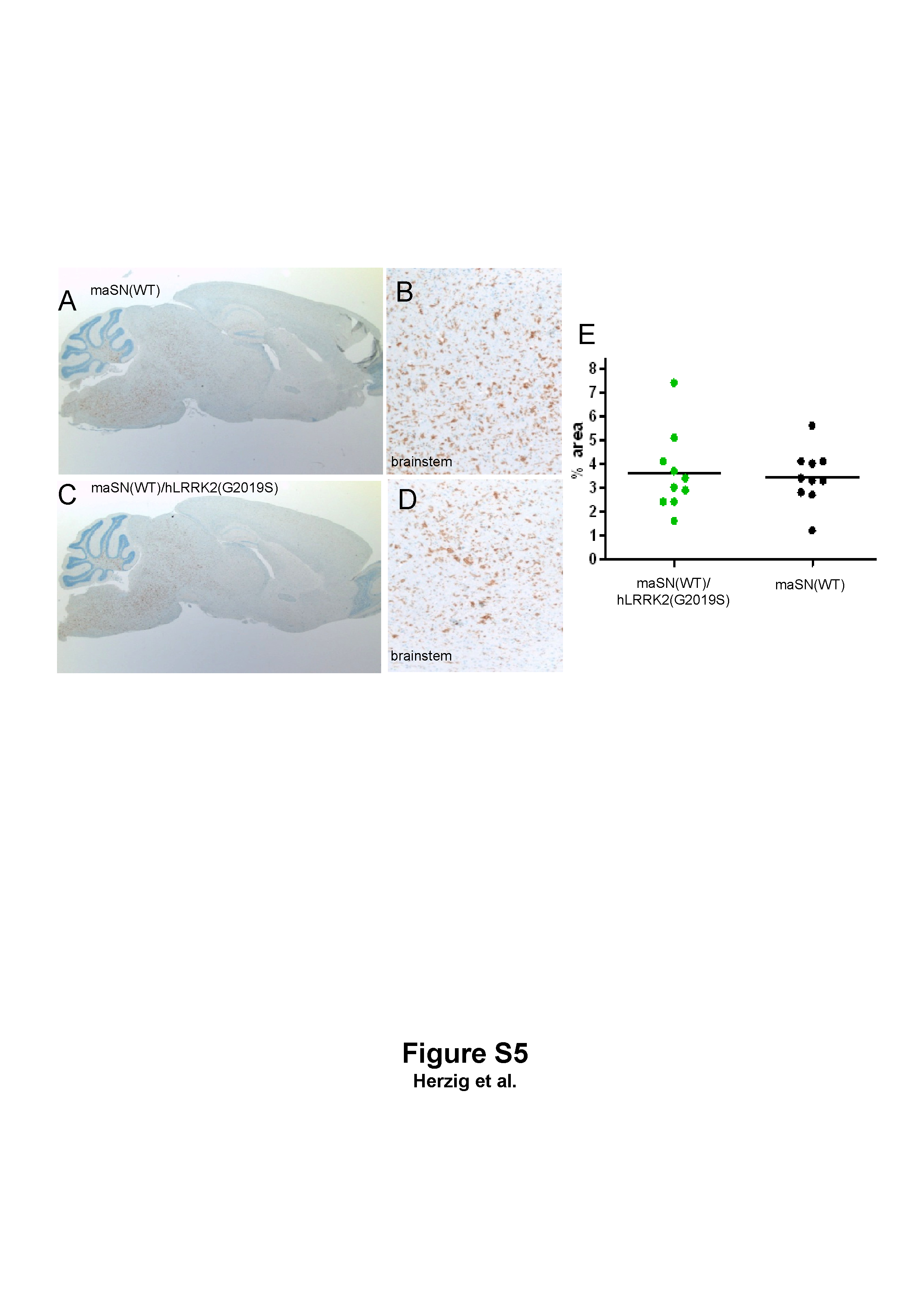

Supplement: Figure S5 — Microgliosis in maSN(WT) and maSN(WT)/hLRRK2(G2019S) end-stage mouse brain. DAB-immunohistochemistry for Iba1 shows activated microglia on a representative sagittal brain section of a maSN(WT) single (A and 20×magnification of brainstem in B) and a haSN(A53T)/hLRRK2(G2019S) double transgenic mouse (C and 20×magnification of brainstem in D). (E) Quantification of the area in the brainstem that is covered by Iba1-positive microglia plotted as % of total area. Dots represent quantifications of individual mice. Control images are shown in Figure 4. (TIF) [file pone.0036581.s005.tif]

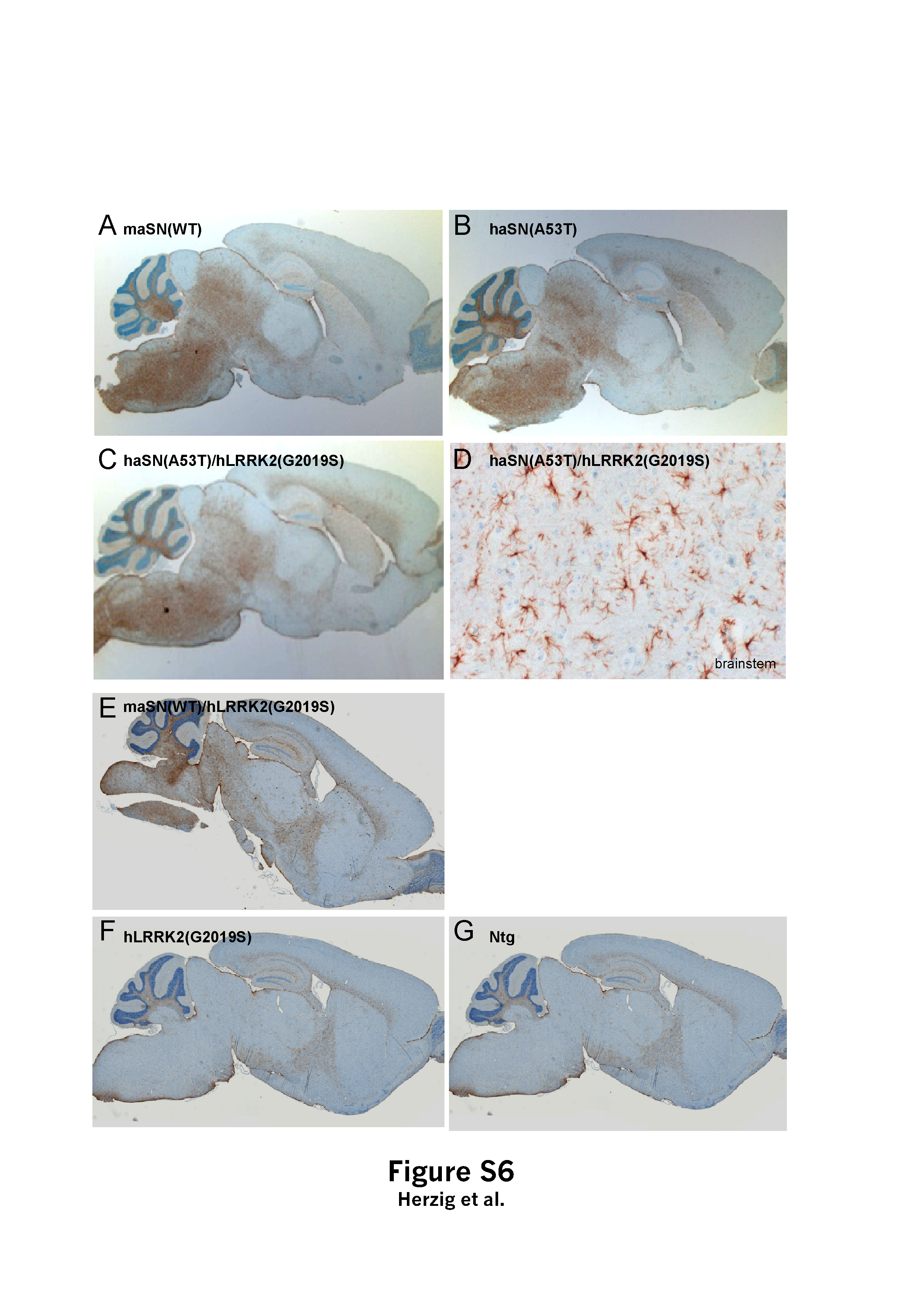

Supplement: Figure S6 — High neuronal levels of LRRK2 do not worsen astrocytosis in haSN(A53T) end-stage mouse brain. DAB-immunohistochemistry for GFAP shows astrocytosis on a represetative sagittal brain section of (A) maSN(WT), (B) haSN(A53T), (C) haSN(A53T)/hLRRK2(G2019S) (20×magnification from brainstem is shown in (D)), (E) maSN(WT)/hLRRK2(G2019S), (F) hLRRK2(G2019S) single transgenic and (G) non-transgenic wildtype littermate control (Ntg) mice. Please note, experiments for (E–G) were performed separately. (TIF) [file pone.0036581.s006.tif]

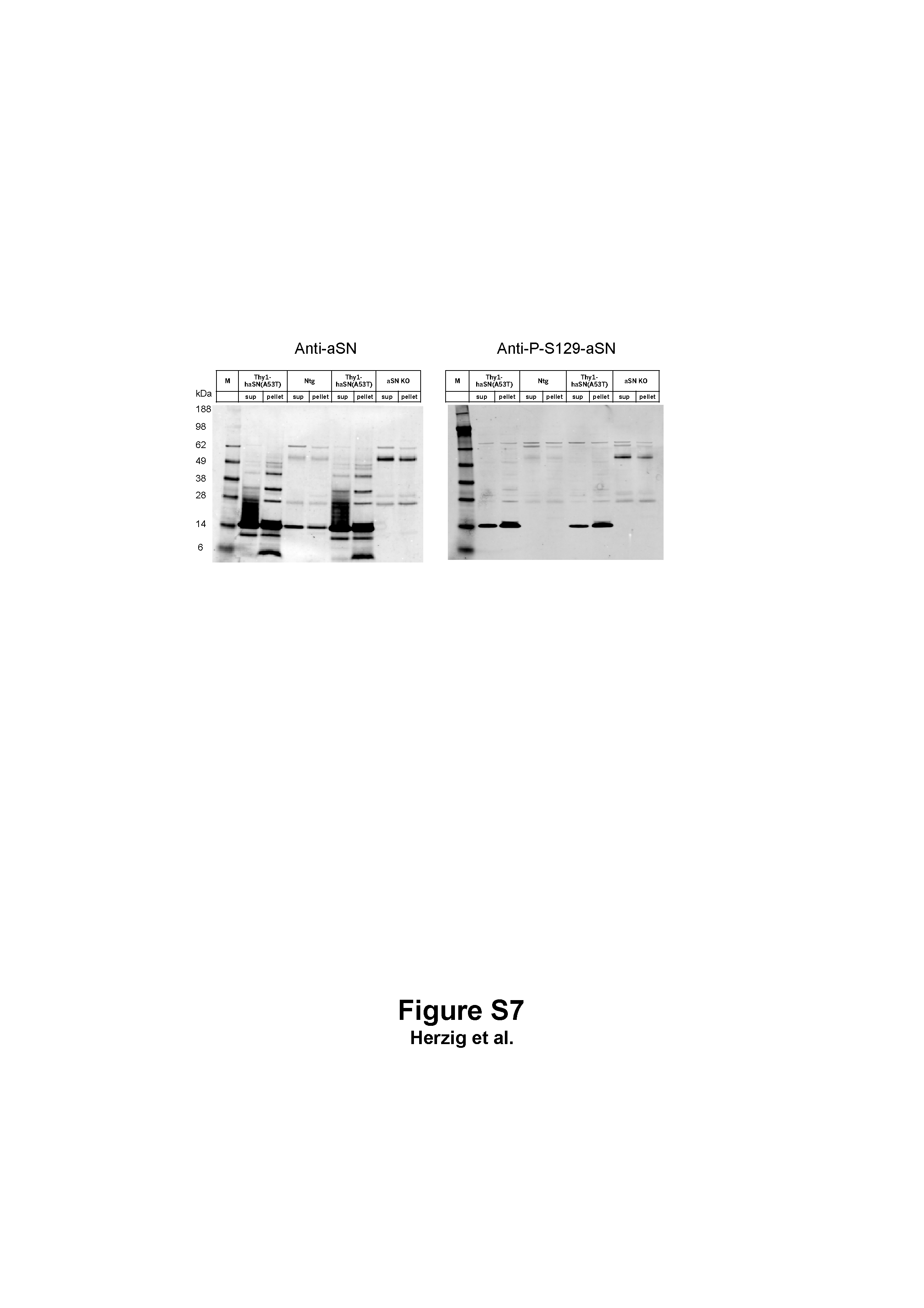

Supplement: Figure S7 — Specificity of aSN antibodies used. Immunoblots of spinal cord extracts from non-transgenic wildtype littermate control (Ntg), haSN(A53T) and aSN knock-out (KO) mice detecting total α-synuclein (aSN), phosphorylated S129-aSN (P-S129-aSN), and unspecific protein species cross-reacting with each antibody (aSN KO lanes). Parts of these results are shown also in Figure 5 for illustration purposes. (TIF) [file pone.0036581.s007.tif]

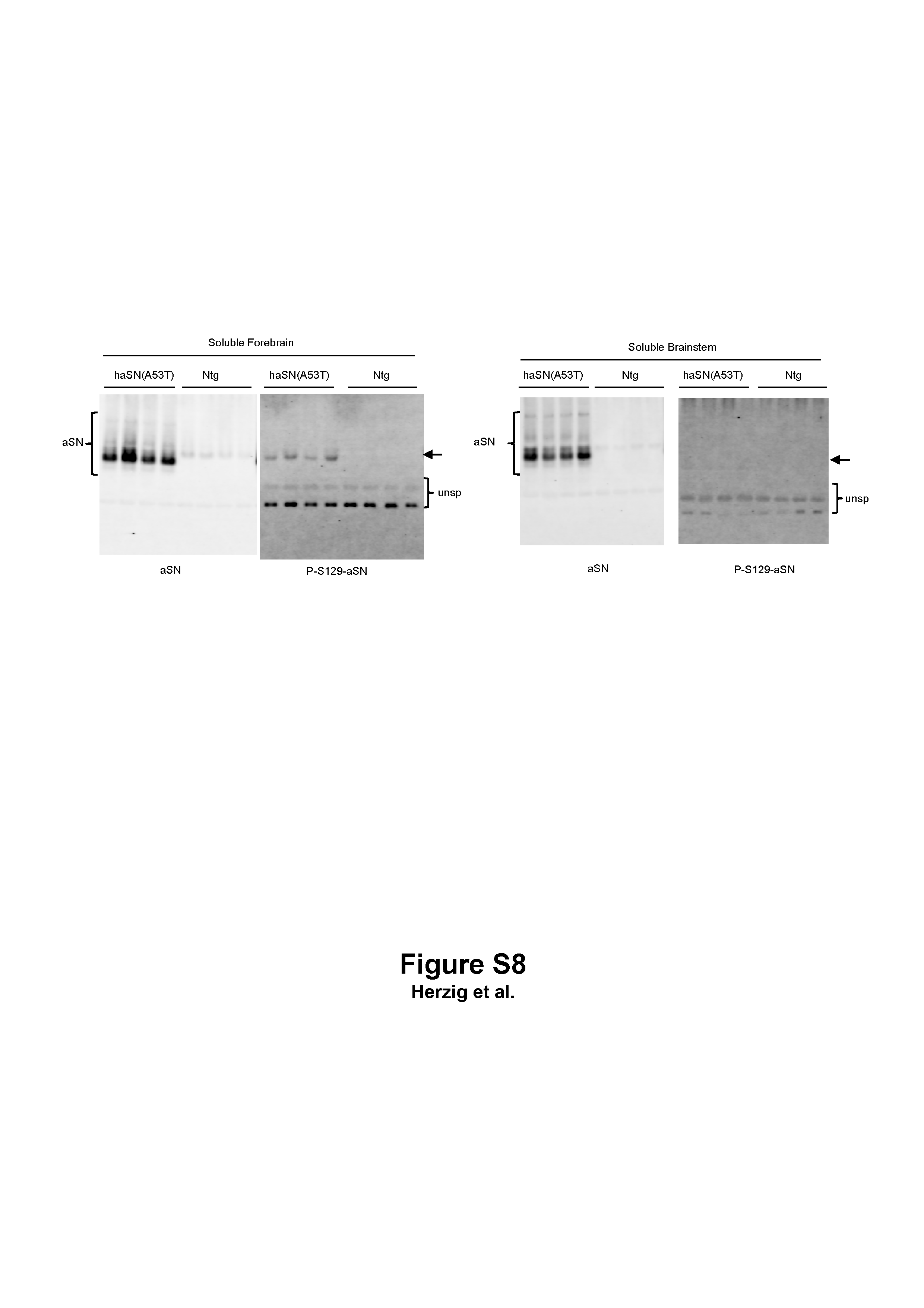

Supplement: Figure S8 — aSN protein species resolved on native gels. Immunoblotting results are shown using antibodies detecting total α-synuclein (aSN) and phosphorylated S129-aSN (P-S129-aSN) (arrows) in soluble protein extracts of forebrain and brainstem comparing non-transgenic wildtype littermate control (Ntg) and haSN(A53T) mice. unsp.: refers to non-aSN proteins cross-reacting with antibody. (TIF) [file pone.0036581.s008.tif]

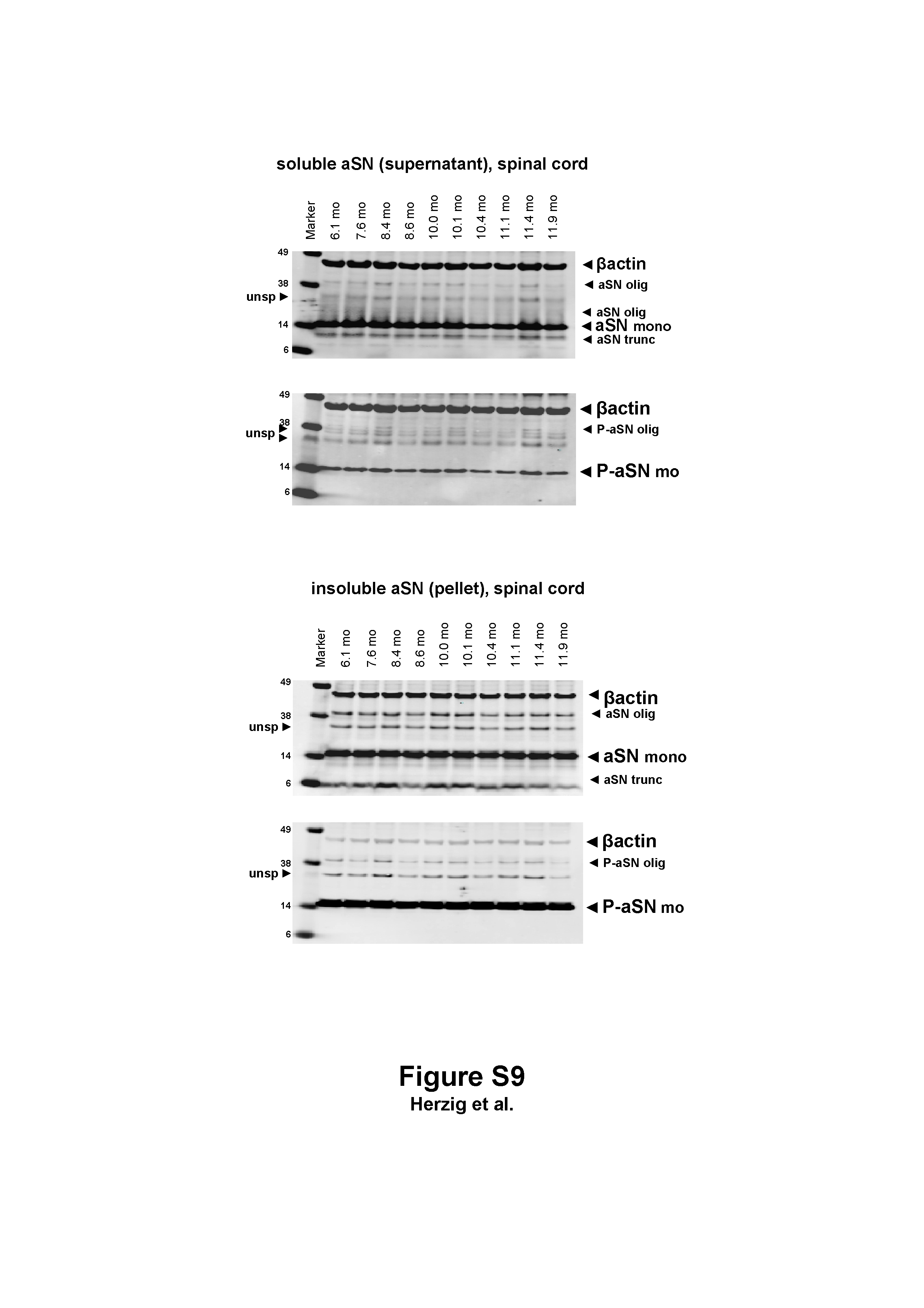

Supplement: Figure S9 — aSN and phospho-S129-aSN levels in spinal cord at different ages of end-stage haSN(A53T)/hLRRK2(G2019S). Immunoblotting of Tris-soluble (supernatant) and Tris-insoluble (pellet) fractions of spinal cord lysates. Blots were stained with antibodies against α-synuclein (αSN) or phosphorylated S129-aSN (pαSN) and β-actin (βactin). The different aSN species are indicated: mono, mo; monomer, olig; oligomer; trunc, truncated. Age indicates the time when each mouse had reached the stage of illness that required us to kill the animal. (TIF) [file pone.0036581.s009.tif]

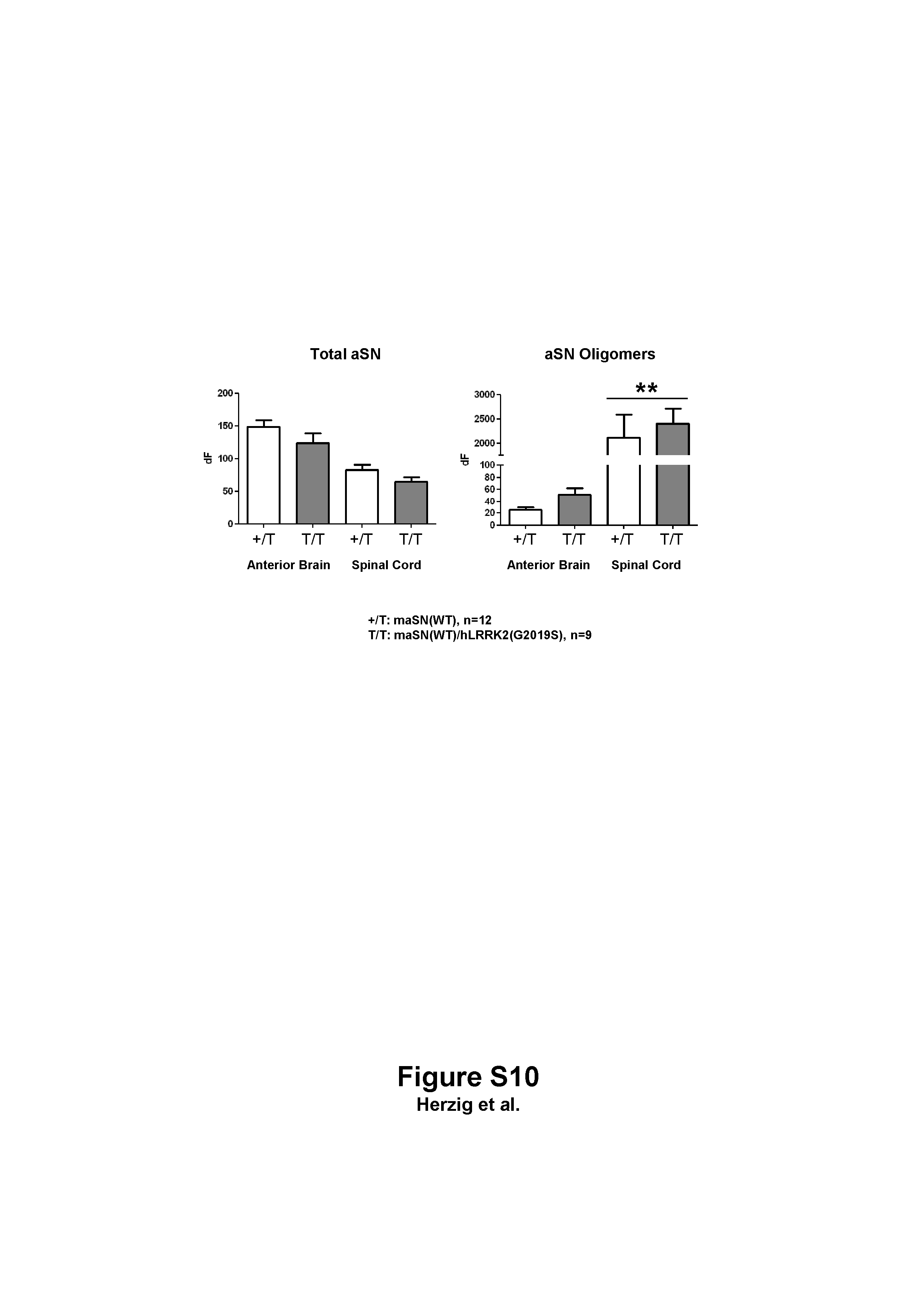

Supplement: Figure S10 — aSN oligomers, detected by TR-FRET, are increased in the spinal cord of end-stage maSN(WT) mice, independently of hLRRK2(G2019S) co-expression. TR-FRET analysis was performed as in Figure 6 on homogenates of the indicated brain regions. Each sample was measured in duplicates. p-values (asterisks indicate p<0.01) were determined by 1way ANOVA (Kruskal-Wallis test). (TIF) [file pone.0036581.s010.tif]

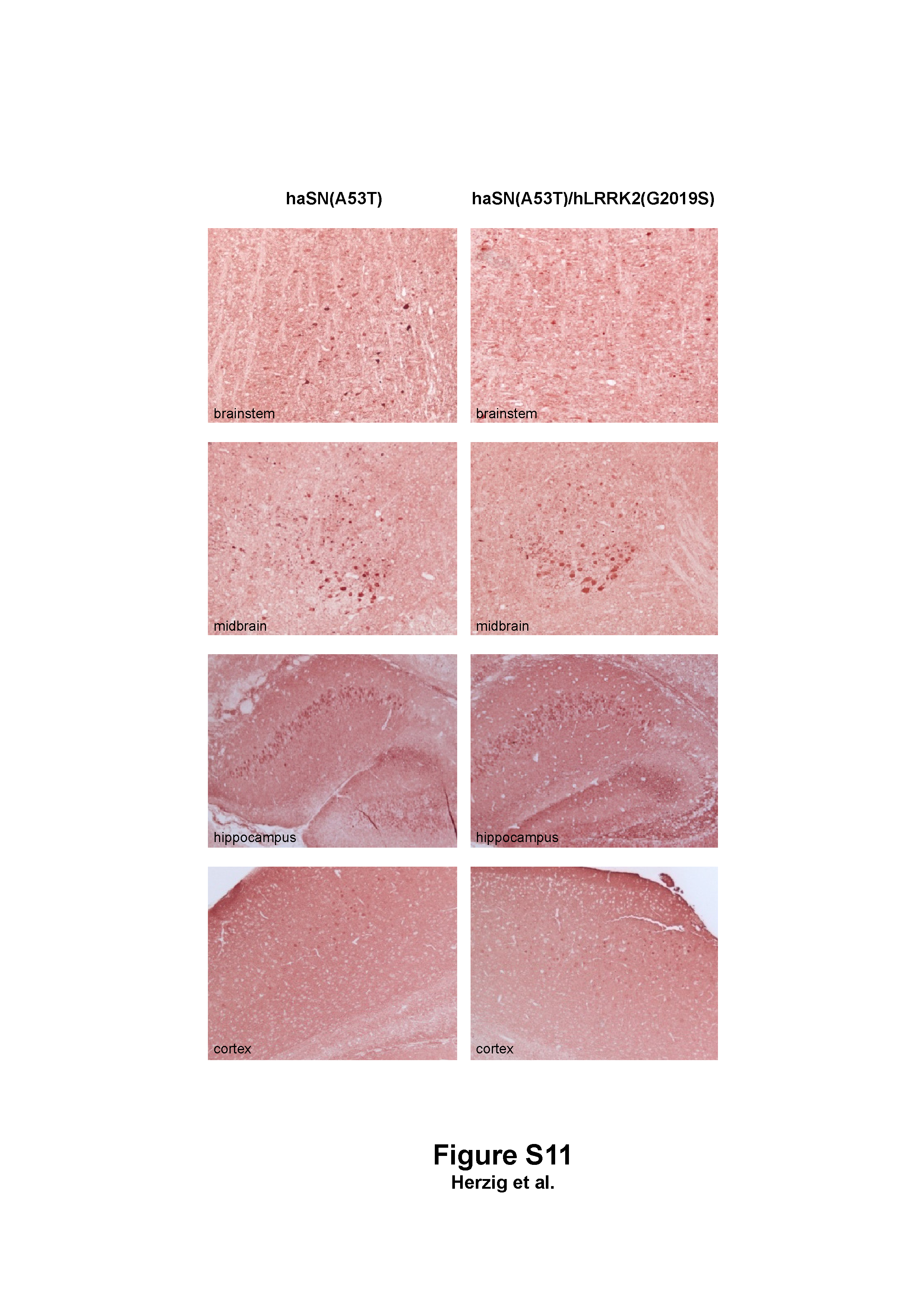

Supplement: Figure S11 — aSN brain histopathology in end-stage haSN(A53T) and haSN(A53T)/hLRRK2(G2019S) mice. For each genotype, a representative section (10×magnification) stained against aSN is shown of the brainstem (reticular formation), midbrain (deep mesencephalic nucleus), hippocampus and cortex (primary motor cortex) of a haSN(A53T) and a haSN(A53T)/hLRRK2(G2019S) mouse. (TIF) [file pone.0036581.s011.tif]

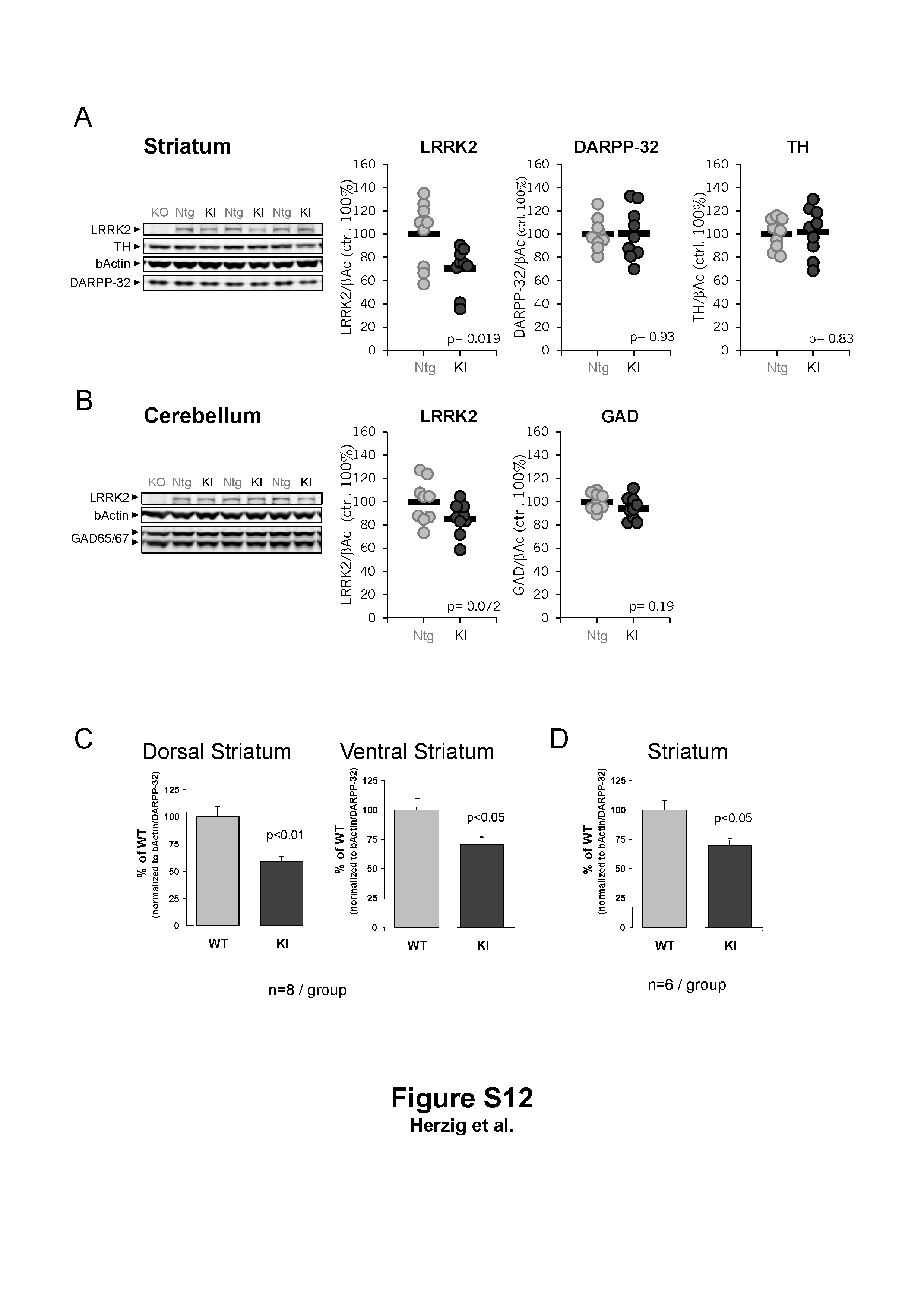

Supplement: Figure S12 — Reduced LRRK2 protein levels in LRRK2(G2019S) knock-in mouse striatum. (A) Immunoblotting results of striatal lysates of Ntg and KI (LRRK2(G2019S) knock-in) female mice are shown (age: 7.5 months). Protein levels determined included LRRK2, DARPP-32 and TH. β-actin (βAc) was used as loading control and for normalization. Circles represent individual mice; the means are indicated as horizontal bars and % are normalized to the protein levels in Ntg. (B) Immunoblotting results for LRRK2 and GAD65/67 of cerebellar lysates from Ntg and KI (LRRK2(G2019S) knock-in) female mice (age: 7.5 months) and quantification of the results. β-actin (βAc) was used as loading control and for normalization. Circles represent individual mice, the means are indicated as horizontal bars and % are normalized to levels in Ntg mice. (C) Quantification of LRRK2 immunoblot results comparing levels in Ntg and KI (LRRK2(G2019S) knock-in) lysates of dorsal, ventral (males, 6.5 months old) and total (dorsal + ventral; females, 5.5 months old) striatum. Values are expressed as % of level in Ntg. Bars indicates SEM. LRRK2 levels were normalized to β-actin and DARPP-32 (which is specifically expressed in the LRRK2-expressing GABAergic projections neurons of the striatum). The number of mice per group is indicated. p-values were determined by two-tailed, unequal variances Student’s t-test. Ntg: non-transgenic wildtype littermate control. (TIF) [file pone.0036581.s012.tif]
